# Genome Wide Association Mapping of Grain and Straw Biomass Traits in the Rice Bengal and Assam Aus Panel (BAAP) Grown Under Alternate Wetting and Drying and Permanently Flooded Irrigation

**DOI:** 10.3389/fpls.2018.01223

**Published:** 2018-09-03

**Authors:** Gareth J. Norton, Anthony J. Travis, Alex Douglas, Susan Fairley, Eduardo De Paiva Alves, Panthita Ruang-areerate, Ma. Elizabeth B. Naredo, Kenneth L. McNally, Mahmud Hossain, Md. Rafiqul Islam, Adam H. Price

**Affiliations:** ^1^School of Biological Sciences, University of Aberdeen, Aberdeen, United Kingdom; ^2^Centre for Genome Enabled Biology and Medicine, University of Aberdeen, Aberdeen, United Kingdom; ^3^National Center for Genetic Engineering and Biotechnology, National Science and Technology Development Agency, Pathum Thani, Thailand; ^4^International Rice Research Institute, Los Baños, Philippines; ^5^Department of Soil Science, Bangladesh Agricultural University, Mymensingh, Bangladesh

**Keywords:** *Oryza sativa*, aus, GWAS, water saving, QTL, yield, harvest index, flowering time

## Abstract

Growing demand for staple crops like rice will need to be achieved predominately through agricultural intensification and more efficient use of inputs. To meet this demand it is essential that the genetic diversity within rice is fully utilized. The *aus* subpopulation is considered an underappreciated resource within that diversity. A new rice panel, the Bengal and Assam Aus Panel (BAAP) of 266 *aus* accessions was generated with ∼2 million informative SNPs obtained using skim sequencing at ∼4× depth. The BAAP was grown in the field in Bangladesh in the ‘boro’ season under both continuously flooded and Alternate Wetting and Drying (AWD) irrigation during 2013 and 2014 in Mymensingh and during 2014 in Madhupur. Heading date, grain mass, straw biomass and harvest index were measured. The majority (94%) of BAAP accessions flowered within a relatively small window of 10 days. The AWD irrigation treatment generally caused an increase in grain mass, but no significant genotype by treatment interactions were detected for this trait. Shoot biomass was the only trait that showed evidence of genotype by treatment interaction. The average LD (Linkage Disequilibrium) decay across the genome was 243 Kbp. Genome wide association mapping revealed 115 quantitative trait loci (QTLs). There was little evidence of QTLs specific to the irrigation treatment, and only a few QTLs co-localized with known genes. However, some QTLs were detected across multiple sites and years. These QTLs should be targets for breeding, and include a region around 2.2 Mbp on chromosome 1, a large region in the middle of chromosome 7 and two regions on chromosome 11 (∼10 Mbp and ∼29 Mbp). The BAAP appears to be a valuable addition to the growing collection of GWA mapping populations of rice.

## Introduction

Rice (*Oryza sativa* L.) is one of the world’s most important cereals as it supplies 35–60% of dietary calorie intake for an estimated three billion people (Fageria, 2007; Global Rice Science Partnership [GRISP], 2013). With an increasing global population, demands on staple crops such as rice will intensify (Mohanty, 2013). Developing new management techniques to increase rice yield and breeding of rice varieties that yield more under a range of different environments, including using inputs more sustainably, is a key target to meet these demands.

Conventional dry season irrigation requires large volumes of freshwater to maintain high yields in rice. It is estimated that to produce 1 kg of rice 2,500 L of water is required (Bouman, 2009); therefore, to produce the average yield of a 4.3 t ha^−1^ in Bangladesh (Global Rice Science Partnership [GRISP], 2013), a total of 10.75 million liters of water is required per hectare. To reduce the volume of water needed for irrigation during the dry season a number of field management techniques have been developed. One method that is being promoted in Bangladesh and other countries is Alternate Wetting and Drying (AWD) (Bouman and Tuong, 2001; Zhang et al., 2009; Lampayan et al., 2015). AWD is a water management technique in which, after plants are established, the field is cycled between flooding and limited field drainage. Once the water table reaches the required depth below the surface (usually in the range of 15–20 cm below the soil surface) the field is re-flooded and the next AWD cycle started. Practitioners of AWD are initially advised not to allow the water level to drop below 15 cm from the soil surface and to stop AWD cycling at the initiation of flowering – this is referred to as “safe-AWD” (Lampayan et al., 2015), but AWD cycling continuing into flowering is also practiced. AWD has been shown to reduce water application by up to 25%. The impacts on yield are variable and are probably related to the degree of soil drying occurring during the AWD cycles and the growth stage at which AWD is applied up to (Carrijo et al., 2017). Safe-AWD appears to have no significant impact, or increase grain yield, whereas more severe AWD treatments can result in a significant reduction in grain yield (Carrijo et al., 2017).

Within rice there is wide natural genetic variation of yield traits (Huang et al., 2010). Traditionally, this variation has been genetically characterized using bi-allelic mapping populations. However, with the advancements in whole genome sequencing the utilization of genome wide association (GWA) mapping has now become common in rice (for example Huang et al., 2010; Zhao et al., 2011; Courtois et al., 2013; Norton et al., 2014; Talukdar et al., 2015; Biscarini et al., 2016; Crowell et al., 2016; Kadam et al., 2017). The most common approach to GWA mapping is to utilize a population of diverse accessions (Zhao et al., 2011). While this approach maximizes the diversity of the alleles (Zhao et al., 2011), and has the potential to identify a larger number of quantitative trait loci (QTLs), there are disadvantages. These include missing rare alleles because of under representation in the GWA population and poor performance of accessions in field environments for which they are not well-adapted (Norton et al., 2014). The latter is particularly problematic as the impact of treatments may differ depending on flowering time (like water management treatments) or if the traits measured are affected by the flowering time of accessions, such as yield or nutrient uptake/distribution. To overcome these issues, GWA populations can be developed utilizing accessions from a geographic or environmentally defined setting provided that the traits of interest are diverse and under strong genetic control within a potentially genetically diverse but restricted population. An example of this approach is given by Biscarini et al. (2016) where GWAS was conducted with a focus on *temperate japonicas* suited to phenotyping in Italy. The population was subsequently assessed under two water regimes, being continuously flooded and a cyclic watering based on a target of −30 kpa soil matric potential (Volante et al., 2017).

Within the global germplasm of rice, the *aus* accessions are a recognized subpopulation (Garris et al., 2005; Travis et al., 2015). Recent sequencing of wild rice relatives suggests that *aus* evolved from a distinct population of the annual *Oryza nivara* found in Bangladesh, Northern Myanmar, and NE India (Kim et al., 2016). Kim et al. (2016) state that “the cultivated *aus* subpopulation and its wild ancestor represent an underappreciated genetic resource.” *Aus* accessions have two attractive features which might make them particularly suitable for GWA mapping. First, they are phenotypically diverse containing the donors of a number of abiotic stress resistance-related traits (Travis et al., 2015). Second, since they are generally considered to be photoperiod insensitive (for adaptation to their normal growing season), many should flower at similar times.

The aim of this study was to develop an *aus* panel for GWA mapping and use it to identify novel genomic loci for yield related traits under continuous flooding (CF) and yield related loci responding to AWD that can be used for breeding. The population was developed using cultivars originally collected from Bangladesh and India. The population was screened for yield traits in the field in Bangladesh at one location over 2 years, and one other location in 1 year. The field trial was conducted using both CF conditions and under AWD. DNA was extracted and sequenced to provide two million SNPs. A large number of loci were identified using GWA mapping of yield component traits, including a number of regions that were stable across treatments, field sites and years, indicating that they are good targets for future study and for plant breeding.

## Materials and Methods

### Development of the Population

The population was designed to contain mostly landraces from the *aus* subpopulation by selecting those reported in Travis et al. (2015) based on similarity (so that two similar cultivars were not included) and flowering time. In addition, 19 of the OryzaSNP set (McNally et al., 2009) plus some released varieties and breeding lines from Bangladesh were included. Among the *aus* cultivars included were 33 present in the Rice Diversity Panel 1 (Zhao et al., 2011) including DJ 123 [the reference *aus* cultivar (Schatz et al., 2014)] and the well-known cultivars N22, Kasalath, FR 13A. The cultivars underwent two rounds of single seed descent at the International Rice Research Institute (IRRI), Philippines. During the second round of single seed decent, DNA was extracted from 298 accessions using Qiagen DNAeasy Plant kits (medium scale) at IRRI and used to conduct whole genome sequencing. Sequencing was conducted by The Centre for Genome Analysis, Norwich, United Kingdom. Twenty DNA samples were pooled for each sequencing lane to allow sequencing on 15 lanes of an Illumina HiSeq 2000 generating 100 bp paired end reads. 299 samples in total were analyzed because one genotype, BRRI Dhan 28 was sequenced twice.

### SNP Calling

The sequencing reads for the 299 DNA samples were cleaned by removing adapters and trimming off low quality bases using Trim Galore!^1^; with parameters -q 20 –length 36 –retain_unpaired. The filtered reads were aligned to the IRGSP-1.0 (International Rice Genome Sequencing Project) reference downloaded from Ensembl v21^2^ using bwa version 0.7.5a with parameters mem –M (Li, 2013). Duplicate reads were marked for removal using Picard Tools version 1.104^3^. Variants for each cultivar were called using GATK version 3.3 HaplotypeCaller (DePristo et al., 2011). A list of known sites for IRGSP-1.0 taken from the 3,000 rice genomes project core SNPs^4^ was filtered to include only cultivars in the Bengal and Assam Aus Panel (BAAP) set using metadata related to subpopulation (Travis et al., 2015). Variants for all cultivars were combined using GATK GenotypeGVCFs resulting in a total of 12,081,601 variants including 10,009,636 bi-allelic SNPs that were filtered on the basis of minor allele frequency (MAF) > 0.05 and <0.1 “missingness” to create a SNP database of 2,053,863 SNPs. The SNP dataset will be available as a project called “BAAP” in the autumn 2018 quarterly update of the SNP-Seek database^5^ (Mansueto et al., 2017) and on the Harvard DataVerse as a data-set “Genome Wide Association mapping of grain and straw biomass traits in the rice Bengal and Assam Aus Panel (BAAP)”^6^.

### A SNP Database, Population Structure, and Linkage Disequilibrium

From the 298 cultivars, only 260 were used to produce an *aus*-specific SNP database. Fifteen of the accessions were OryzaSNP accessions from other subpopulations (eight OryzaSNP accessions being *japonicas* and seven *indicas*), while there were eight Bangladeshi *japonicas* and 10 Bangladeshi *indicas*. In addition, five *aus* landraces had sequence depth lower than 1× and were eliminated, while one had high heterozygosity and was eliminated (see **Supplementary File S1** for details).

Population structure analysis was conducted using fastSTRUCTURE (Raj et al., 2014*)* and SNPhylo (Lee et al., 2014). An examination of the results from fastSTRUCTURE and the neighbor-joining tree (not shown) suggest there is significant population structure present with up to five sub-groups in the BAAP panel and this was taken into account in the GWAS analysis.

Global LD (Linkage Disequilibrium) was calculated as *R*^2^ using PLINK (Purcell et al., 2007) with parameters “–r2 dprime –ld-window-kb 5000 –ld-window 99999 –ld-window-r2 0.” Global LD decay was defined as the distance at which *R*^2^ < 0.2, estimated by binning LD values for each chromosome.

### Field Screening

The population was screened in Bangladesh under both AWD and continuously flooded (CF) conditions in 2013 and 2014 in Mymensingh, and in 2014 in Madhupur. The full details of the field screening is given in Norton et al. (2017a,b). Briefly, for the field screen in 2013 at Mymensingh rice seeds were sown in a nursery bed on 31st December 2012. The day before transplanting the seedlings into the experimental plots, the plots were fertilized with 40 kg ha^−1^ nitrogen, 20 kg ha^−1^ phosphorus, 70 kg ha^−1^ potassium, 15 kg ha^−1^ sulfur, and 3 kg ha^−1^ zinc. A further 40 kg ha^−1^ nitrogen was supplied during the tillering stage (26th March), and another 40 kg ha^−1^ nitrogen at the flowering stage (6th April). The seedlings were transplanted into the eight plots (each plot was 5 m × 11.4 m) on the 13th of February 2013. They were planted as two plants per hill with a distance of 20 cm between each hill in a row and a 20 cm distance between each row of 4 m length. At total of 276 and 282 (CF and AWD, respectively) rice accessions were planted in single rows, with a check cultivar BRRI Dhan 28 transplanted into each alternate row. After transplanting the plots were flooded. For the four CF plots the surface water was kept at a depth of between 2 cm and 5 cm above the soil surface during the vegetative and reproductive stages (13th April 2013). For the four AWD plots plastic perforated tubes (pani pipe) were placed across the plots to monitor the water depth. The aim was to allow water to drain/percolate naturally from the AWD plots until the average depth of the water was 15 cm below the soil surface. At that point the plots were irrigated to bring the water depth to between 2 cm and 5 cm above the soil surface. Both the AWD and CF plots were kept under the same flooded conditions up until 3rd March when water was withheld from the AWD plots for the start of the first AWD cycle. There were four AWD cycles with the fourth finishing on the 11th April. At this point the rice plants had started flowering and the AWD plots were kept flooded and maintained the same as the CF plots.

Once the cultivars had flowered and the grain matured, the grain and shoots from every cultivar was hand harvested from the six central hills of each row. The grain was then hand threshed and weighed to determine the grain mass per six hills (referred to as grain mass). The shoots were harvested approximately 5 cm above the soil, dried, and then weighed to determine the shoot weight per six hills (referred to as straw biomass).

For the field experiment at Mymensingh in 2014 the rice seeds were sown in a nursery bed on 17th December 2013. The field site was prepared as described for 2013, with the rice plants transplanted on 6th February into the eight plots (each plot was 22.7 m × 11.8 m). A total of 254 and 257 (CF and AWD, respectively) accessions were planted out at this field site. The fertilizer regime was as for 2013, with the split application of nitrogen fertilizer applied on 27th February and 27th March. The AWD cycles for the four AWD plots started on the 11th of February with the fourth cycle ending on 10th April. Once the fourth cycle had finished, the AWD and CF plots were maintained under flooded conditions during the flowering stage. Rice cultivars were harvested as described above.

For the field experiment at Madhupur in 2014 the rice seeds were sown in a nursery bed at Mymensingh on the 17th December 2013. The field site was prepared as described above for the Mymensingh site. The seedlings were transplanted into the eight plots at Madhupur on the 8th and 9th February. Each plot was 24 m × 10 m. Plants were planted as two plants per hill with a distance of 20 cm between each plant in a row and 20 cm distance per row. A total of 271 and 273 (CF and AWD, respectively) accessions were planted out at this field site. The fertilizer regime was as for 2013, with the split application of nitrogen fertilizer applied on 1st March and 30th March. The AWD cycles for the four AWD plots started on the 3rd March with the fourth cycle ending on 19th April. Once the fourth cycle had finished, the AWD and CF plots were maintained under flooded conditions during the flowering stage. Rice cultivars were harvested as described above.

In all the field experiments for each treatment a randomized complete block design was employed with four replicate blocks, but treatments were separated (see Norton et al., 2017a,b for full details). Further analyses (GWAS, ANOVA, and correlations) were only conducted when data was available for at least three replicates. The exception to this rule was flowering time which was measured on only one replicate block because experience informed us that variation between replicates for flowering time was negligible. For example, measuring flowering time of 114 members of the Rice Diversity Panel 1 in India with four replicates revealed genotype explained 99.92% of the variation (essentially 100% heritability) rendering the need for replication redundant (Tapash Dasgupta, University of Calcutta, unpublished data).

### Population Structure

Population structure was analyzed using STRUCTURE (Pritchard et al., 2000; Falush et al., 2003) and STRUCTURE Harvester (Earl and vonHoldt, 2012) as described by Travis et al. (2015). The number of distinct population sub-groups was estimated using the Evanno Delta-K method (Evanno et al., 2005). Although STRUCTURE is well-suited to analysis of 326 SNP markers, fastSTRUCTURE (Raj et al., 2014) and ADMIXTURE (Alexander et al., 2009) implement more efficient population models better suited to analyze the 2,053,863 SNP markers obtained in this study. The greater resolution of 2,053,863 SNP markers allowed more population groups to be identified by fastSTRUCTURE, using marginal likelihood, as model complexity was increased. The number of population sub-groups was also identified using ADMIXTURE (Alexander et al., 2009) using CV (Cross-Validation), as model complexity was increased. An 80% threshold of group membership was used to classify cultivars into population sub-groups.

### Genome Wide Association Mapping

Genome wide association mapping was performed using PIQUE (Parallel Identification of QTL’s Using EMMAX^7^) to pre-process genotype and phenotype data for subsequent analysis by EMMAX (Kang et al., 2010) followed by EMMAX analyses on each phenotype in parallel (GitHub repo for PIQUE). A mixed effects model was used to estimate the association between each SNP and phenotype across all cultivars, whilst accounting for population structure and cryptic kinship. For the fixed effects, population structure was included as covariates based on the first five principal components of PCA of all 2,053,863 BAAP SNPs across cultivars using EIGENSTRAT smartpca (Price et al., 2006). Random effects were estimated using a kinship matrix to measure the genetic similarity between individuals as the proportion of times a given pair of cultivars had the same genotype across all SNPs (IBS values). Information about population structure and kinship was incorporated into GWAS models across all cultivars as both fixed and random effects. The false discovery rate (FDR) of detected associations was estimated using the R-language Bioconductor “multtest” library to calculate Benjamini–Hochberg adjusted probabilities (Benjamini and Hochberg, 1995). A significance threshold of 10% FDR was used to identify putative SNP associations (McCouch et al., 2016).

After GWA, SNPs with −log_10_(P) < 4 were examined to group the SNPs into QTL. If two SNPs were closer than the genome average LD decay value of 250 Kbp, they were considered to belong to the same locus.

### Statistical Analysis

All statistical analyses were performed using the statistical software Minitab v.17 (State College, PA, United States) and SigmaPlot v.13 (Systat Software Inc., San Jose, CA, United States) and the significance of ANOVA main effects and interactions reported at alpha < 0.05. Assumptions of normality were tested using the Anderson–Darling test in Minitab. For the plant mass traits two-way ANOVA was conducted with AWD and CF, and cultivar and interaction terms as the explanatory variables for each site. For the 2013 Mymensingh field site there were a total of 221 genotypes in common across both treatments. At Mymensingh in 2014 a total of 232 genotypes were common across both treatments. At Madhupur in 2014 a total of 226 genotypes were common across both treatments. For the plant mass traits three-way ANOVA was conducted with treatment (AWD and CF), site and cultivar (common across all sites *n* = 191) as the explanatory variables. For the three-way ANOVA the presence of an interaction between the three explanatory variables was also determined. For correlation analysis a Pearson’s correlation was used.

## Results

### The BAAP – Genome Sequencing, SNP Database, Linkage Disequilibrium (LD), and Population Structure

The BAAP population developed in this study (detailed in **Supplementary File S1**) was selected from a larger panel of 511 rice cultivars screened by Travis et al. (2015). From the cultivars screened in that study, a total of 300 were selected for the BAAP, including 266 landraces identified as belonging to the *aus* subpopulation. The selected *aus* cultivars flower within a window of 79–92 days. The 326 SNPs reported in Travis et al. (2015) were used to avoid selection of very closely related cultivars. In addition to these *aus* cultivars, the panel also contains the OryzaSNP cultivars (McNally et al., 2009) and a number of elite Bangladeshi cultivars.

A total of 298 cultivars from the BAAP population were sequenced to coverage depths ranging from 0.42 to 37×, with an average of 5.5×. To create the SNP database only 266 accessions from the *aus* subpopulation were used, which had a sequencing depth of above 1.40×. Sequence reads were aligned to the Nipponbare reference and SNPs were then called, imputed in regions of low coverage and filtered to give a total of 2,053,863 SNPs detected across the BAAP population (an average of 1 SNP per 210 bp).

**FIGURE 1:**
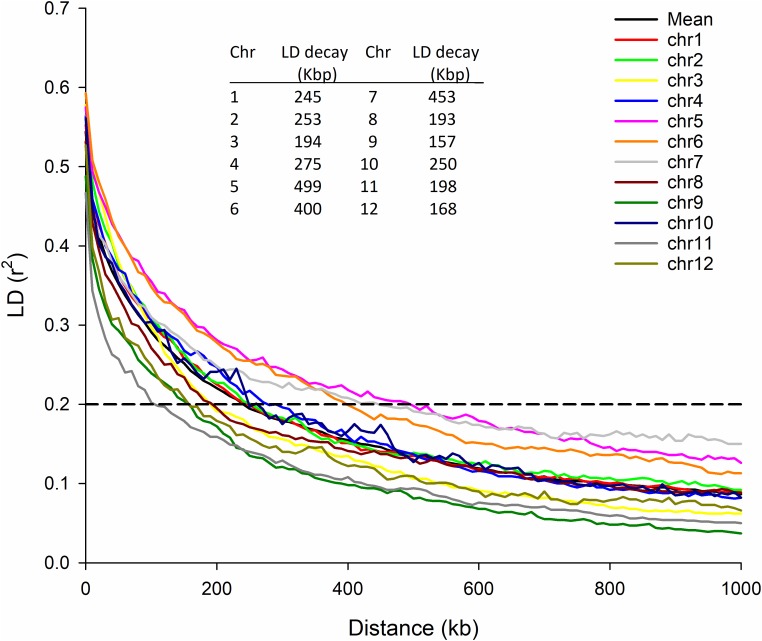
LD decay by chromosome. LD plots for each chromosome and the mean LD for all 12 chromosomes. Insert is the distance (Kbp) where LD drops below r2 of 0.2 for each chromosome.

The average LD decayed across the entire rice genome for these cultivars at 243 Kbp, but was not uniform across chromosomes. The lowest per-chromosome LD decay was observed on chromosome 9 (157 Kbp) and the largest on chromosome 5 (499 Kbp) (**Figure 1**).

**FIGURE 2:**
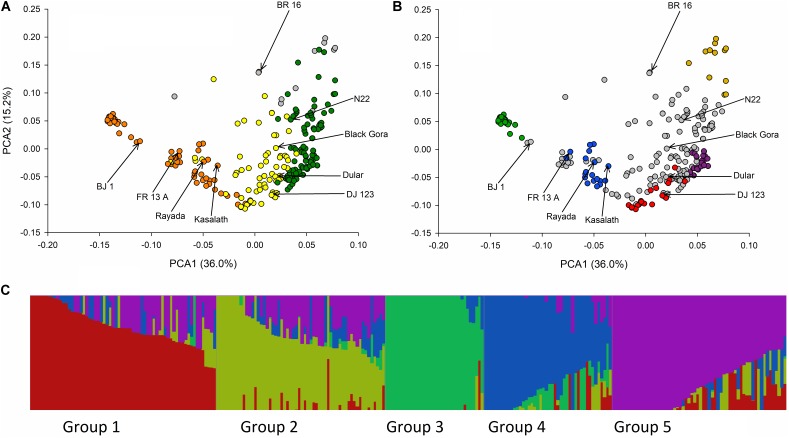
Principle components and fastSTRUCTURE analysis of BAAP. **(A)** Biplot of the first two PCA axis of the 266 *aus* accessions of the BAAP using 2,053,863 SNPs color coded according to the classification of population groups of Travis et al. (2015) based on 326 SNPs where orange is “aus-1,” green is “aus-2,” yellow is “aus-admix,” and gray is “admix.” **(B)** Same PCA biplot color coded according to *K* = 5 fastSTRUCTURE subgroups where group 1 is red, group 2 is yellow, group 3 is green, group 4 is blue, group 5 is purple, and accessions that do not fall into these groups are gray. **(C)** Distruct (Rosenberg, 2004) plot of fastSTRUCTURE subpopulation membership coefficients with *K* = 5.

Analysis of population structure in 326 SNP markers across the entire genome of 511 rice cultivars from which the BAAP was selected revealed four distinct subpopulation groups, these being an *indica* group, a *japonica* group and two *aus* groups (Travis et al., 2015). Analysis of the population structure using the same 326 SNPs for the 266 BAAP subset of the 511 cultivars revealed five distinct populations. The number of population groups identified by fastSTRUCTURE using all markers are shown in **Supplementary Figure S2**. The marginal likelihood curve begins to plateau at *K* = 5 population subgroups (**Supplementary Figure S2**), consistent with the results obtained using STRUCTURE on the BAAP for 326 SNPs (**Supplementary Figure S1**). Similarly, the number of population groups identified by ADMIXTURE using CV (Cross-Validation) begins to plateau at *K* = 5 population subgroups (**Supplementary Figure S3**). Assuming there are five population subgroups, based on the output of fastSTRUCTURE the 260 cultivars used to make the SNP database fall into group 1 (21 cultivars including DJ 123), group 2 (13 cultivars), group 3 (29 cultivars), group 4 (21 cultivars including FR13A Kasalath and Rayada), group 5 (30 cultivars) and admix (146 cultivars including BJ1, Black Gora, Dular and N22) (**Supplementary File S1** and **Figure 2**). Considered of note is the observation that groups 1, 3, and 5 are predominantly from Bangladesh (71%, 86%, and 90%, respectively) while groups 2 and 4 are predominantly from India (92% and 76%). Also considered noteworthy is that of the 21 cultivars which have the term “boro” in their name, 20 belong to group 3. All 20 are from Bangladesh. The cultivar Boro Black is in group 4 and is from India.

The first two axes of the PCA of 2,053.863 SNPs are shown in **Figure 2** and reveal a clear separation of the BAAP cultivars into different population groups consistent with the classification previously used by Travis et al. (2015) (**Figure 2A**) and that revealed by fastSTRUCTURE (**Figure 2B**). The five fastSTRUCTURE groups revealed here map onto those revealed by Travis et al. (2015) as follows; groups 3 and 4 are Travis aus-1, groups 2 and 5 are Travis aus-2 while group 1 was aus-admix in Travis et al. (2015). The Distruct (Rosenberg, 2004) plot of subpopulation membership coefficients with *K* = 5 is shown in **Figure 2C**.

### Field Screening

The panel was screened in Bangladesh at Mymensingh in 2013 and 2014, as well as Madhupur in 2014, under both CF (Continuously Flooded) and AWD (Alternate Wetting and Drying) irrigation. A summary of the population means for all traits measured is given in **Table 1**.

**Table 1:** Descriptive statistics of the traits for the genotypes grown under each treatment.

Site	Year	Trait	Treatment	Mean	*SD*	Min	Median	Max
Mymensingh	2013	Grain mass (g)	AWD	80.8	20.7	25.2	79.7	140.9
			CF	60.5	17.6	22.7	59.2	120.2
		Straw biomass (g)	AWD	111.0	28.0	57.6	106.5	260.5
			CF	100.8	24.5	53.3	97.9	199.2
		Harvest index	AWD	0.421	0.003	0.157	0.432	0.508
			CF	0.373	0.003	0.048	0.210	0.477
		Flowering time (d)	AWD	101.3	3.9	96.0	100.0	130.0
			CF	102.9	4.6	96.0	102.0	126.0
	2014	Grain mass (g)	AWD	46.6	16.1	10.8	44.5	108.0
			CF	39.5	17.6	8.0	37.4	112.3
		Straw biomass (g)	AWD	79.1	27.0	26.0	72.8	167.8
			CF	83.6	35.8	18.5	76.0	197.8
		Harvest index	AWD	0.370	0.053	0.185	0.374	0.518
			CF	0.320	0.053	0.132	0.319	0.455
		Flowering time (d)	AWD	115.8	4.0	107.0	115.0	127.0
			CF	116.8	3.7	110.0	117.0	129.0
Madhupur	2014	Grain mass (g)	AWD	56.36	18.01	18.0	53.00	127.0
			CF	50.28	17.81	14.5	47.63	117.8
		Straw biomass (g)	AWD	93.66	32.15	42.25	86.00	185.0
			CF	92.35	33.02	30.75	83.00	213.8
		Harvest index	AWD	0.377	0.048	0.177	0.385	0.465
			CF	0.352	0.048	0.203	0.354	0.484

*Values are average per hill from six central hills within each replicate row of test cultivars (two plants per hill). Only data from the 266 aus cultivars are presented.*

**FIGURE 3:**
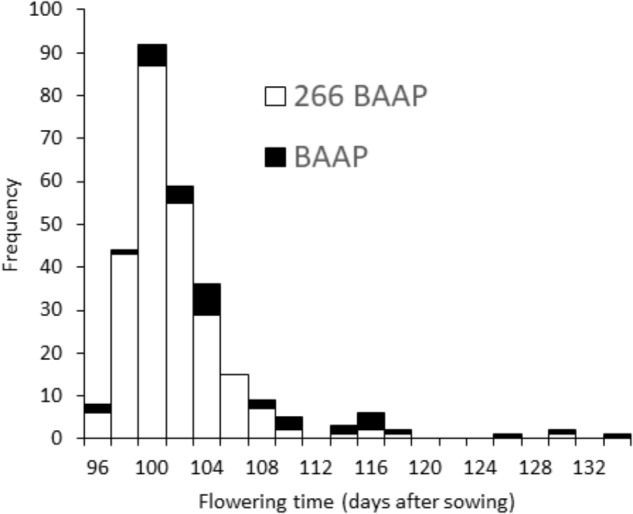
Flowering time distribution. Frequency distribution of flowering time in AWD Mymensingh 2013, showing the whole panel (black) and just the 266 *aus* cultivars (white).

The initial strategy was to have all members of the population to initiate flowering within a 10-day window. However, when grown in the field in Bangladesh, the initiation of flowering from first to last cultivar was longer than this (**Table 1**), and in the most extreme scenario (the AWD experiment in year 1), the flowering initiation window was 34 days (see histogram presented in **Figure 3**). Yet, the proportion of cultivars that initiated flowering 5 days either side of the mean for each experiment was between 83.2 and 94.4% of the cultivars.

**FIGURE 4:**
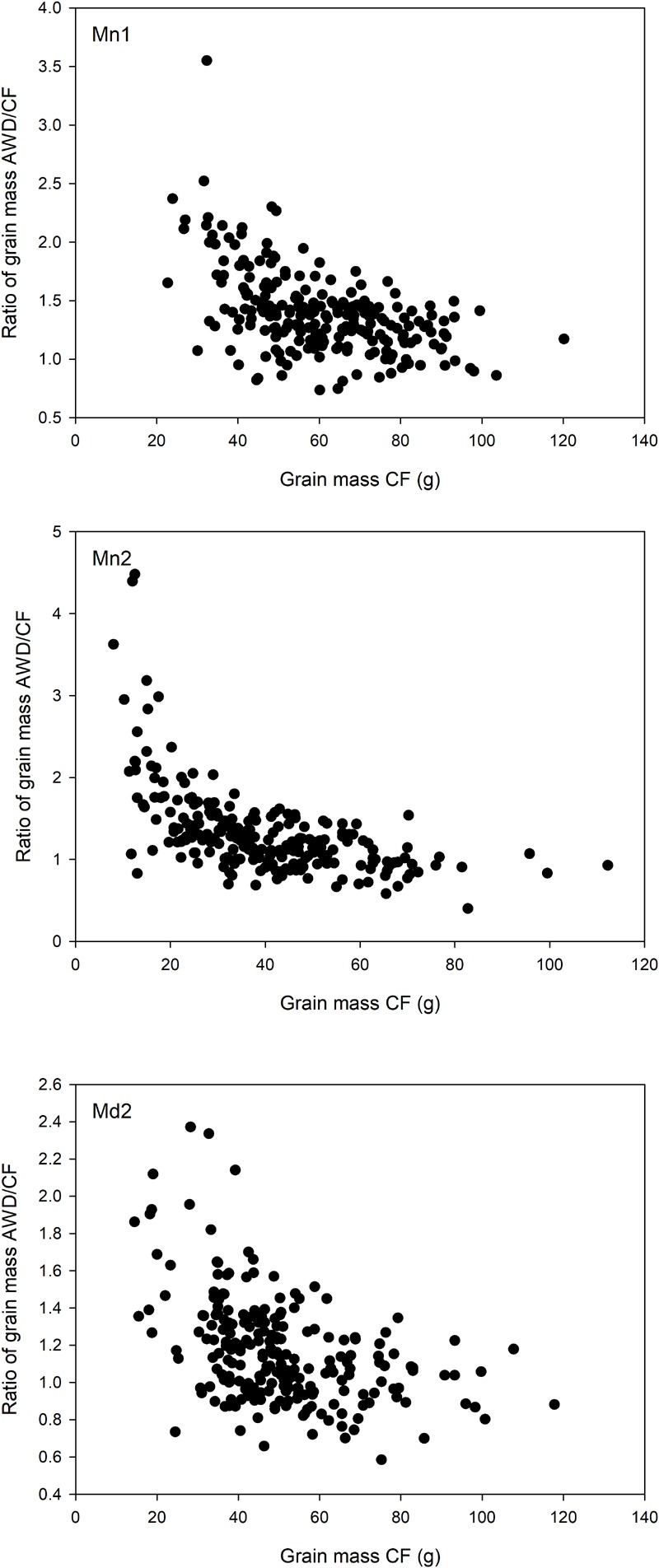
Responsiveness to AWD. Relationship between grain mass in the cultivars grown under CF conditions and the ratio of grain mass in the cultivars grown in AWD:CF at the Mymensingh site year 1 **(Top)**, Mymensingh year 2 **(Middle)**, and at Madhupur year 2 **(Bottom)**. Points are the mean of three or four replicates.

Overall, grain yield, straw biomass and harvest index was highest in Mymensingh 2013 and lowest in Mymensingh 2014. There was large variation between cultivars for all traits measured. Two-way ANOVA conducted for each site separately revealed strong effects of genotype, generally weaker effects of AWD treatment and little genotype by treatment interactions (**Table 2**). Means of each treatment and the correlations between treatments for each site are presented in **Supplementary Figures S4** (grain mass), **S5** (straw biomass), **S6** (harvest index), and **S7** (flowering time). AWD had mostly positive impacts on plant growth and partition traits. For grain mass, treatment explained 1.8–11.9% of variation with AWD producing higher grain mass. There was, however, no genotype by treatment interaction. The impact of AWD was smaller on straw biomass (not significant in Madhupur), but there was a genotype by treatment interaction at two sites explaining approximately 6% of the variation. The impact of AWD was stronger on harvest index. Correlations between the same trait under AWD or CF conditions were generally very strong (and highly significant), with the correlation coefficient ranging from 0.68 to 0.79 for grain mass, 0.73 to 0.86 for straw biomass, 0.46 to 0.59 for HI, 0.65–0.76 for FT (**Supplementary Figures S4**–**S7**). Correlations performed on 191 cultivars for grain mass, straw biomass, and harvest index between years and sites within the same treatment are presented in **Supplementary Figures S8**–**S10**. For grain mass in all cases the correlations were highly significant (r ranged from 0.485 to 0.749; **Supplementary Figure S8**). When plotting the grain mass produced for each cultivar against the AWD responsiveness ratio (ratio of grain mass produced when grown under AWD vs. grain mass produced when grown under CF), it can be observed that the cultivars with the highest ratio (i.e., produce a much greater grain mass under AWD compared to CF) are predominantly the cultivars that produce less grain mass under CF (**Figure 4**). There was, however, no correlation between sites for this responsiveness to AWD ratio.

**Table 2:** Statistical analysis of traits at each site.

Site	Year	Trait	Grain mass	Straw biomass	Harvest index
Mymensingh	2013^1^	Treatment	328 ^∗∗∗^	65.8 ^∗∗∗^	222.2 ^∗∗∗^
			(11.9%)	(2.0%)	(9.4%)
		Genotype	4.27 ^∗∗∗^	7.16^∗∗∗^	2.89 ^∗∗∗^
			(34.2%)	(49.5%)	(26.7%)
		Treatment × genotype	NS	NS	NS
	2014^2^	Treatment	89.66 ^∗∗∗^	10.9 ^∗∗∗^	208.6 ^∗∗∗^
			(2.6%)	(0.3%)	(8.4%)
		Genotype	7.09 ^∗∗∗^	12.9 ^∗∗∗^	2.87 ^∗∗∗^
			(49.7%)	(64.2%)	(26.8%)
		Treatment × genotype	NS	1.17 ^∗^	NS
				(6.0%)	
Madhupur	2014^3^	Treatment	65.22 ^∗∗∗^	NS	97.16 ^∗∗∗^
			(1.8%)		(3.3%)
		Genotype	8.95 ^∗∗∗^	13.64 ^∗∗∗^	5.03 ^∗∗∗^
			(55.0%)	(65.6%)	(39.5%)
		Treatment × genotype	NS	1.18 ^∗^	1.29 ^∗∗^
				(5.9%)	(10.1%)

*Values reported are F-values from the ANOVA and the values in brackets is the percentage contribution of the factor to the observed variation. ^1^At Mymensingh in 2013 a total of 221 genotypes were common across both treatments. ^2^At Mymensingh in 2014 a total of 232 genotypes were common across both treatments. ^3^At Madhupur in 2014 a total of 226 genotypes were common across both treatments. ^∗^p < 0.05; ^∗∗^p < 0.01; ^∗∗∗^p < 0.001.*

An import aspect of understanding how AWD impacts upon different rice cultivars compared to CF, is to determine if these responses are variable under different environments irrespective of treatment (i.e., field sites). A three way ANOVA, which included field site, revealed genotypes explaining the greatest proportion of the variation in grain and straw biomass and harvest index (**Table 3**) with site explaining the next highest proportion of the variation and then treatment explaining less variation. There was evidence of significant field site by treatment (i.e., the impact that the treatments have is different in different environments) and field site by genotype (i.e., cultivars respond differently in different environments) interaction with the latter explaining 7.6–9.5% of the variation. Only for straw biomass did this analysis reveal genotype by treatment interaction explaining 2.1% of the variation.

**Table 3:** Three-way ANOVA of the traits for the cultivars common across all three field sites (*n* = 191).

	Trait		
Factor/interaction	Grain mass	Straw biomass	Harvest index
Field site (F)	820.45^∗∗∗^	333.53^∗∗∗^	261.60^∗∗∗^
	(17.53%)	(5.91%)	(7.55%)
Treatment (T)	397.00^∗∗∗^	6.84^∗∗^	490.46^∗∗∗^
	(4.24%)	(0.06%)	(7.12%)
Genotype (G)	13.49^∗∗∗^	28.62^∗∗∗^	6.74^∗∗∗^
	(27.51%)	(48.29%)	(18.71%)
F × T	66.76^∗∗∗^	33.60^∗∗∗^	22.01^∗∗∗^
	(1.45%)	(0.62%)	(0.65%)
F × G	1.87^∗∗∗^	2.82^∗∗∗^	1.38^∗∗∗^
	(7.60%)	(9.50%)	(7.60%)
T × G	NS	1.26^∗^	NS
		(2.14%)	
F × T × G	NS	NS	NS

*Values reported are F-values from the ANOVA and the values in brackets is the percentage contribution of the factor to the observed variation. ^∗^p < 0.05; ^∗∗^p < 0.01; ^∗∗∗^p < 0.001.*

### Genome Wide Association Mapping of Yield-Related Traits

**FIGURE 5:**
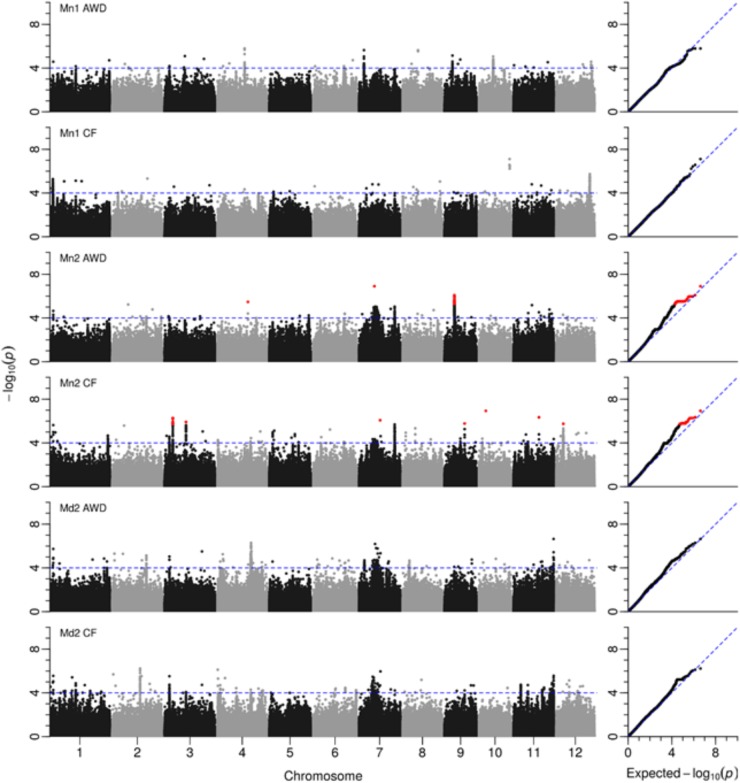
GWAS for grain yield. Manhattan and QQ plots from GWA mapping of grain yield in field experiments under AWD (Alternate Wetting and Drying) and CF (Continuous Flooding) over year 1 and 2 at sites in Mymensingh (Mn1, Mn2) and year 2 in Madhupur (Md2), Bangladesh. A guide-line in blue is shown at –log10(0.0001) = 4. Benjamini–Hochberg adjusted probabilities > 0.1 are highlighted in red. The diagonal blue line shown on the QQ plots represents 1:1 agreement between expected probability, according to a null hypothesis, and the observed probability of SNP association.

A total of 2,720 SNPs were significantly associated with grain yield in at least one of the six environments tested. **Figure 5** presents the Manhattan plots while **Supplementary File S2** provides a summary of every SNP where –log_10_(P) < 4 with minor allele frequency and effect size, plus a graph for each chromosome. A total of 32 loci were notable where there were either multiple significant SNPs associated with one field in at least one environment, or SNPs associated with multiple fields. These loci were located; one on chromosome 5, two on chromosomes 2, 6, 8, 9, and 12, three on chromosomes 1 and 3, four on chromosomes 4 and 7 and five on chromosome 11 (**Table 4**). Nine of these putative QTLs were detected in only one environment while 14 were detected in two. Only the QTL around 2.2 Mbp on chromosome 1 was detected in all six environments, while the QTLs detected around 3.45 Mbp on chromosome 3, and in the center of chromosome 7 were detected in five environments. It must be noted that there are two very broad QTLs in the center of chromosome 7 spanning 8.76–11.90 Mbp and 12.30–14.61 Mbp that are reported here as two separate QTLs detected in all environments except Mymensingh year 1 AWD. It may be these represent one very broad QTL.

**Table 4:** Notable association detected for yield.

		Number of significant SNPs detected					
		Mn Year 1		Mn Year 2		Md Year 2	
Chromosome	Position (Mbp)	AWD	CF	AWD	CF	AWD	CF
1	2.17–2.43	1	71	4	4	5	5
1	18.65–18.67	1	1				11
1	41.47–41.86				4		
2	20.00–20.19			1			84
2	24.48–24.51			3		12	
3	3.46–3.47	3		1	7	3	10
3	5.88–5.90				48		
3	15.39–15.43				40		1
4	0.13–0.42				6		3
4	1.89–2.05					2	6
4	19.37–19.51	27	1				
4	23.63–24.42				4	425	3
5	2.34–2.45				12	1	
6	22.4–23.04					1	8
6	30.84–30.85						8
7	3.85–3.86					34	
7	8.76–11.90^∗^		2	93	11	14	43
7	12.30–14.61^∗^		1	44	2	18	8
7	25.63–25.79			42	44		1
8	4.23–4.87					9	
8	11.08	3	2				
9	6.94–7.34			688		1	
9	14.42				6		1
10	10.16–10.34	248					
10	21.97–22.22		4				
11	5.25						15
11	10.34–10.38				1		3
11	20.59–20.83					3	19
11	27.70–27.80					1	9
11	28.76–28.99				1	2	4
12	4.76–5.13				1	241	1
12	24.07–24.19		95				

*^∗^Two regions on chromosome 7 are very broad, containing a continuum of significant SNPs.*

A total of 8,399 SNPs were associated with straw biomass in at least one of the six environments tested (**Supplementary Figure S11** and **Supplementary File S3**), revealing 59 notable loci where there were multiple significant SNPs associated with the trait in at least one environment. These loci were located: one on chromosome 10, two on chromosomes 9, three on chromosomes 2, 5, and 12, four on chromosomes 6 and 8, five on chromosome 1, six on chromosome 3 and ten on both chromosomes 7 and 11 (**Supplementary Table S1**). Twelve of these QTLs were detected in only one environment while 19 were detected in two, 11 were detected in 3, 8 were detected in 4, 7 in 5 leaving 2 QTLs detected in all six environments. These two ubiquitous QTLs were both on chromosome 7 around 15 Mbp and 25.6 Mbp. As with yield, it must be noted that there is a very broad region of QTL activity from 9.35 to 16.93 Mbp where it is not clear how many QTLs are present. Four are reported here, but it might be one very wide one. Eighteen of the straw biomass QTLs detected here overlap with QTLs for yield. Eight of the loci have been previously reported as QTLs for straw biomass from Liu et al. (2006), Suji et al. (2012), and Bhattarai and Subudhi (2018) (full details in **Supplementary Table S1**).

A total of 1,853 SNPs were associated with harvest index in at least one environment (**Supplementary Figure S12** and **Supplementary File S4**) revealing 35 notable loci. There were nine loci detected on chromosome 11, four on chromosomes 1 and 9, three on chromosomes 2, 3, 6, and 12, two on chromosomes 5 and 8, one on chromosome 10 and none on chromosome 4. No loci were detected in all environments or even in 5/6 but two were detected in four environments being around 20.25 on chromosome 9 and 19.5 on chromosome 11. Four of the harvest index QTLs detected are in the same place as yield QTLs being at 2.2 Mbp on chromosome 1, 3.85 Mbp on chromosome 7 and both 20.7 and 22.8 Mbp on chromosome 11 (**Supplementary Table S2**). Two of the QTLs detected here were also detected using GWA by Guo et al. (2018), one was detected as a QTL by Hittalmani et al. (2003), and one co-localizes with the ABERRANT PANICLE ORGANIZATION 1 (*APO1*) gene identified at 27.5 Mb on chromosome 6 by Terao et al. (2010) (full details in **Supplementary Table S2**).

### Genome Wide Association Mapping of Flowering Time

**FIGURE 6:**
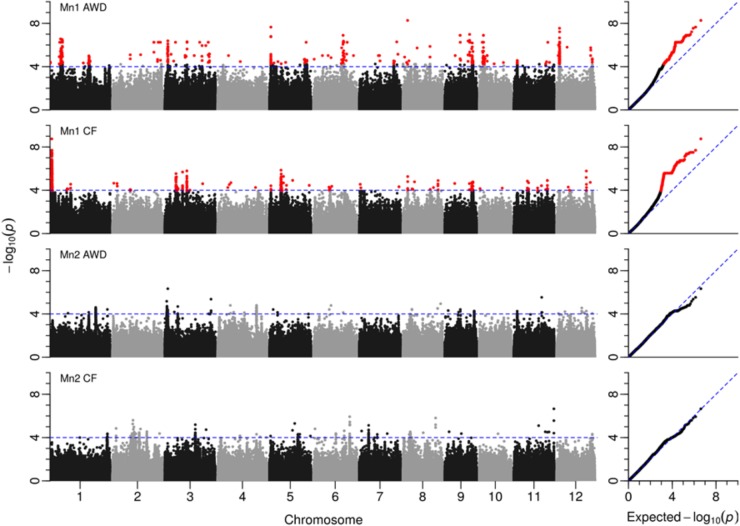
GWAS for flowering time. Manhattan plots from GWA mapping flowering time (for details, see **Figure 6**).

Across the genome and over the four experiments where flowering time was recorded, a total of 3,515 SNPs were significantly associated with flowering time (**Figure 6** and **Supplementary File S5**). A total of 26 loci were detected that either had a number of significant SNPs co-localized from a single experiment or where SNPs from multiple experiments co-localize (**Table 5**). Of the 26 loci highlighted, in 10 instances significant SNPs were co-localized from two experiments and in only a single instance were SNPs from three experiments co-localized.

**Table 5:** Notable association detected for flowering time in Mymensingh.

		Number of significant SNPs detected			
		Year 1		Year 2	
Chromosome	Position (Mbp)	AWD	CF	AWD	CF
1	1.167–1.683		1729		
1	7.848–8.069	458			
1	27.894–28.870	79		1	
1	32.853–32.921			31	
1	41.307–41.318			1	11
3	1.524–1.630			113	
3	2.145–2.196	4		6	
3	7.911–8.072		24		
3	12.732–12.811		9		
3	15.757–15.904		19		
3	21.738–22.010				40
4	27.788–27.881			105	1
5	0.992–1.026	53	2		
5	8.143–8.510	1	90		
6	22.064–22.067	3			2
6	25.996–26.061				37
7	6.690–6.718				16
7	24.770–24.789	11			
8	5.211–5.229				12
8	19.427–19.616	10	1		
9	19.521–19.683	28	4		
10	2.900–3.780	84			
11	2.393–2.510	32		4	
11	28.607–28.866	2			3
12	1.879–2.346	185			
12	25.663–25.739	11		1	2

In addition to the identification of loci across the genome, the identification of SNPs within LD of known candidate genes involved in flowering time in rice was investigated (**Table 6**). A conservative estimate of genome wide LD of 200 Kbp was used. Unlike the identification of QTLs for flowering time, where only notable QTLs were reported (**Table 5**), all significant SNPs were considered when identifying SNPS that were located near known (200 Kbp) flowering time genes. Significant SNPs were identified within the estimated LD for a total of 8 previously identified genes involved in flowering in rice (**Table 6**), with two of the genes (Hd5 and RCN) having significant SNPs from more than one screen being within the candidate region.

**Table 6:** Co-localization of significant SNPs with genes and loci believed to be involved in flowering time in rice.

Flowering time gene/loci	Trait	LOC^1^	Ch	Mbp^2^	number of SNPs within LD^3^
OsCRY2*^a^*	CF year 2	Os02g41550	2	24.92	2
OsMADS50*^b^*	AWD year 2	Os03g03100	3	1.30	6
OsDof12^c^	AWD year 1	Os03g07360	3	3.74	18
Hd16*^d^*	AWD year 2	Os03g57940	3	33.00	1
Hd1^e^	CF year 1	Os06g19444	6	11.07	2
Ehd3*^f^*	AWD year 1	Os08g01420	8	0.27	1
Hd5/DTH8/Ghd8/LHD1/Ds9*^g^*	AWD year 1	Os08g07740	8	4.33	1
Hd5/DTH8/Ghd8/LHD1/Ds9*^g^*	CF year 2	Os08g07740	8	4.33	1
RCN1*^h^*	AWD year 1	Os11g05470	11	2.45	32
RCN1*^h^*	AWD year 2	Os11g05470	11	2.45	4

*^1^Locus name based on MSU 7.0. ^2^Indicates the start of the gene in MSU 7.0. ^3^LD is estimated to be 200 Kbp for each gene. ^a^Hirose et al., 2006; ^b^Lee et al., 2004; ^c^Li et al., 2009; ^d^Hori et al., 2013; ^e^Yano et al., 2000; ^f^Matsubara et al., 2011; ^g^Wei et al., 2010; ^h^Nakagawa et al., 2002.*

### Combining All QTLs

**FIGURE 7:**
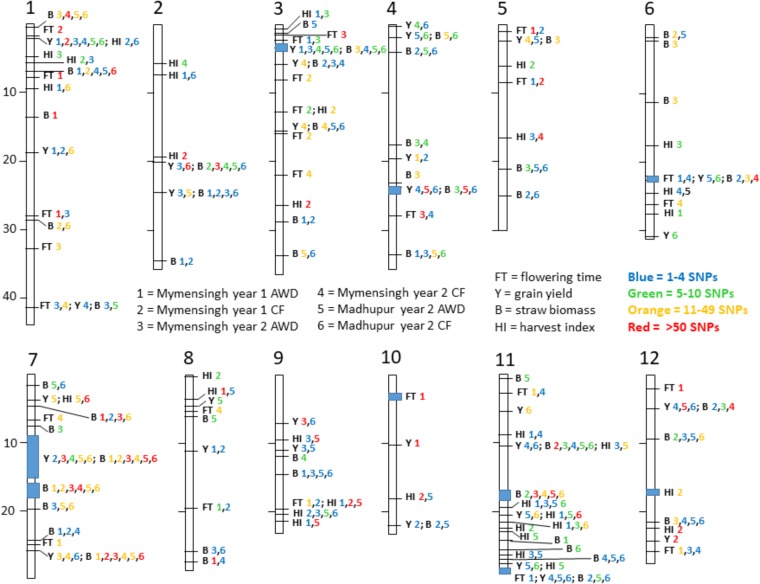
Summary of loci. Map of all notable loci detected for flowering time and yield-related traits. Each trait has a one or two letter identifier according to the central legend. Each site is numbered and the number is color coded to reflect the number of SNPs detected in that site. For example, at the very top of chromosome 1 is indicated B 1,4,5,6 where three numbers are orange and the 4 is red. This means in this locus between 11 and 49 significant SNPs were detected in Mymensingh year 1 AWD, and both AWD and CF in Madhupur in year 2 while there were more than 50 SNPs detected in Mymensingh year 2 CF, all for straw biomass. In eight locations there is a blue box indicating the spread of SNPs over a large area making it difficult to discern if it is one broad QTL or multiple close loci.

The fact that 115 loci were detected across all traits highlights the genetic complexity of the traits under study. In order to demonstrate a pattern is clearly observed, **Table 7** presents a list of loci that are notable because both grain mass and straw mass QTLs coincide, while a summary of the all the QTLs detected for the traits is presented in **Figure 7**. In order to identify QTLs based on GWA results, SNPs were considered to be from the same locus if they were within 250 Kbp of each other. While this resulted mostly in small regions containing multiple “significant” SNPs, often for multiple traits, there were nine regions where this approach produced larger clusters which may reflect the occurrence of either multiple underlying QTLs or low local LD decay. These are marked as blue blocks in **Figure 7**. The two blocks on chromosome 7 are particularly broad each being several Mbp, suggesting they cannot represent conventional single QTLs.

**Table 7:** Notable QTLs detected for grain mass and straw biomass that co-localize where A = AWD treatment and C = CF treatment, Mn1 = Mymensingh 2013, Mn2 = Mymensingh 2013, and Md2 = Madhupur 2014.

Loci		Grain mass			Straw biomass		
Ch	Mbp	Mn1	Mn2	Md2	Mn1	Mn2	Md2
2	20		A	C	C	A, C	A, C
2	24.5		A	A	A, C	A	C
3	3.0–3.5	A	A, C	A, C		A, C	A, C
4	24		C	A, C		A	A, C
6	22.7			A, C	C	A, C	
7	14.8	C	A, C	A, C	A, C	A, C	A, C
7	25.7		A, C	C	A, C	A, C	A, C
11	10.3		C	C	C	A, C	A, C
11	28.8		C	A, C	C		A, C
12	5.0		C	A, C	C	A, C	

Quantitative trait loci considered particularly noteworthy are (i) around 2.3 Mbp on chromosome 1 yield QTLs were detected in all environments, and harvest index in two of the environments, (ii) between 3.0 and 3.5 Mbp on chromosome 3 where yield QTLs were detected in five environments and biomass in four, (iii) a broad region on chromosome 7 centered on about 15.0 Mbp where there are QTLs for yield and biomass for, respectively, five and six environments, (iv) around 26 Mbp on chromosome 7 where there are biomass QTLs in all environments and yield in three, and (v) around 10.3 on chromosome 11 where there are straw biomass QTLs in five environments, yield in two and harvest index in two. There were three loci where there were multiple yield QTLs which co-localized with flowering time suggesting a role for the timing of flowering in determining yield: 41 Mbp on chromosome 1, 22 Mbp on chromosome 6 and 29 Mbp on chromosome 11. None of these loci are at known flowering time genes.

## Discussion

### The BAAP

The objective in creating the BAAP was to produce a population with little population structure, good adaptation to the target environment (i.e., was geographically adapted) and having genetic variation for traits of interest. Structure analysis of the BAAP population identified a number of subgroups (five or more) within the *aus* used in this study. This is in contrast to previous studies that identified two subgroups within the *aus* subpopulation (Travis et al., 2015) and presumably reflects the greater power to detect structure based on orders of magnitude more makers. The population structure does match, however, that detected in Travis et al. (2015) which can clearly be seen comparing **Figure 2A** with **Figure 2B**. Groups 3 and 4 reported here correspond to *aus*-1 reported in Travis et al. (2015) while groups 2 and 5 correspond to *aus*-2. Group 3 reported here seems particularly noteworthy since it is predominantly Bangladesh cultivars and contains all but one of the cultivars with the term “boro” in its name. Since boro refers to the dry (winter) growing season in Bangladesh (Travis et al., 2015), this suggests group 3 cultivars have been selected for suitability to irrigated boro production in Bangladesh.

The average LD decay within the population was 243 Kbp which is similar to the approximate 200 Kbp reported for *aus* in Zhao et al. (2011). There was more than twofold variation in average LD decay between chromosomes being below 200 Kbp for chromosomes 3, 8, 9, 11, and 12 and 400 Kbp or above for chromosomes 5, 6, and 7.

While this association population has reduced genetic diversity compared global rice association populations [e.g., the RDP1 (Zhao et al., 2011)], it has increased genetic diversity with a single subgroup, and wide phenotypic variation for the traits measured in this study. The fold range for grain mass ranged from 5.3 to 14.0 across the six experiments, while the fold range for straw biomass was 3.7 – 10.7 across the experiments (**Table 1**). Not only was a high degree of variability in the phenotypes observed, the contribution of genotype to the observed variation was high (**Tables 2**, **3**).

One of the aims in the generation of this population was to have a reduced flowering window. During flowering initiation and grain filling environmental factors could have an impact on grain production and grain quality. Therefore if the window of flowering initiation is limited to short period, the cultivars are more likely to be undergoing similar environmental responses during this key developmental stage. A majority (83.2–94.4%) of the cultivars flowered within a 10-day window. This general coincidence of flowering between accessions is better than other association populations, for example the RDP1 which displayed a 20-day window for 90% of the population to flower when grown in Faridpur, Bangladesh or 40 days when grown in Arkansas, United States (Zhao et al., 2011).

### Impact of AWD

As previously identified in a subset of these cultivars (Norton et al., 2017a,b), in this study AWD increased grain mass. For the whole population AWD increased grain mass in Mymensingh 2013, Mymensingh 2014, and Madhupur 2014 by 32.2%, 18.0%, and 12.1%, respectively (**Table 1**). The highest yielding cultivars under AWD were BJ 1 (the cultivar with the highest yield in both Mymensingh year 1 and Madhupur, and the third highest yield in Mymensingh year 2), and Jagle Boro (the cultivar with the highest yield in Mymensingh year 2 and fifth highest yield in Mymensingh year 2). Other notable high yielding cultivars under AWD include Kasalath and Pachodi 427.

A recent meta-analysis on the impacts of AWD on yield has been conducted (Carrijo et al., 2017), the overall findings being that AWD caused a reduction in yields, by on average 5.4%. However, when the AWD treatments were broken down into the degree of water treatment severity, methods that implemented a mild AWD (when soil water potential was ≥20 kPa or if field water level did not drop below 15 cm from the soils surface) the meta-analysis concluded that there was no negative impacts on yield (Carrijo et al., 2017). In this study (which by using the criteria of Carrijo et al. (2017) would be described as a mild AWD treatment) we observed that grain yield is increased under mild AWD in agreement with other studies (Yang et al., 2009, 2017; Zhang et al., 2009; Wang et al., 2014). The reason for this increase in yield is yet unknown. Novel to this study is the wide range of cultivars that underwent the same AWD treatment, however the lack of strong genotype by treatment interactions for the yield traits revealed by ANOVA suggests that there is limited breeding potential for adaption to AWD and that cultivars generally increased in yield when exposed to the AWD treatment. On the other hand, it is notable that there is evidence of genotype by treatment interaction for straw biomass which might merit more study, and it should also be recognized that the experimental design with only four replicates is not well suited to detect anything but strong genotype by treatment interaction.

In contrast to the above, correlation analysis might offer a different interpretation of the importance of genotype by AWD interaction. The average increases in grain mass observed in this study are some of the highest increases in production reported for AWD. A potential reason for this could be the genetic background of these cultivars. In many studies the cultivars selected for field trails are those that are already high yielding cultivars and therefore potential management methods to increase yield have limited impact, while in this study the cultivars selected are not generally high yield cultivars. This means that the AWD treatment could have caused the cultivars to get closer to their potential yield. This is supported by the observation of an increase in grain mass of 9.0% and 9.4% (Mymensingh 2013 and 2014, respectively) for the high yield Boro cultivar, BRRI dhan28, grown in the same experiment (Norton et al., 2017a) compared to the average yield increase for the population of 32.2% and 18.0% (Mymensingh 2013 and 2014, respectively) when grown under AWD. It was observed that some of the highest increases in percentage yield (higher in AWD than CF) were for the cultivars that had some of the lowest grain yield in CF (**Figure 3**). The cultivars from the *aus* subpopulation of rice are a diverse set of cultivars and have been shown to have tolerances to a wide range of environmental scenarios including submergence tolerance (Xu et al., 2006), phosphorus starvation tolerance (Gamuyao et al., 2012), drought resistance (Henry et al., 2011), and heat tolerance (Jagadish et al., 2008). It is possible that they are less well adapted to flooding than the improved cultivars generally used in the Boro season. Many of these landraces might be upland cultivars although this information is not available. Hence, the fact that there is a substantial increase in grain mass for some cultivars under AWD, may not be that AWD causes an increase in yield in these cultivars, but rather that CF causes a reduction in the cultivars’ yield potential. Thus, there is evidence of cultivar by genotype interaction, but since this indicates that the highest yielders in AWD are those that already yield well in CF, the results still do not suggest breeding for AWD is a priority for research.

Volante et al. (2017) have used GWA mapping to study a locally adapted rice panel grown under continuous flooding and “low water” treatment which was a form of AWD. Differences between their study and this are in the subpopulation used (*temperate japonica* vs. *aus*), the degree of potential water stress experience by the plants (−30 kPa at 20 cm soil depth vs. −15 cm water depth), the number of SNPs available (31 K vs. 2 M), and the traits measured (multiple physiological, morphological and yield components vs. yield and biomass). It is important to note that only flowering time can be directly compared across experiments and especially that in their study all traits were negatively affected by the water treatment indicating it was on the severe side of recommended treatments for AWD. Unlike the current study, about half of the QTLs detected by Volante et al. (2017) appeared to be treatment specific. This may reflect the relatively harsher water stress applied (in current study there was no negative impact of AWD). One QTL was detected in both studies, for days to maturity in Volante et al. (2017) on chromosome 4 at 27 Mbp detected in low water treatment only which was detected strongly in the current study under AWD in year 2 with just 1 SNP detected in the CF for that year. It is possible this is a gene affecting flowering under water stress.

### QTLs and Candidate Genes

In this study, a large number of QTLs were detected for all the measured traits. Of particular interest are the QTLs for grain mass and straw biomass that are stable, i.e., detected in multiple experiments and conditions (**Figure 7** and **Table 7**). There are a total of 10 loci, where grain mass and/or straw biomass QTLS appear to be co-localized on numerous occasions, many of these QTLs are detected across years and sites. This indicates that these are stable QTLs and ideal targets for plant breeding. Some of the most interesting loci are those at 2.3 Mbp on chromosome 1, the middle of chromosome 7, and at both 10.3 and 28.8 Mbp on chromosome 11.

The genetic basis of yield has been reviewed by Liang et al. (2016b), where they report 39 QTLs either cloned or tagged which effect yield in rice. Because of the availability of this review, comparisons with the QTLs detected for the BAAP were made. Only three loci detected here are within 250 Kbp of these genes/QTLs, being *Gnp4* at 19.6 Mbp on chromosome 4, both Ghd7 at 9.1 and qSPP7 at 10.0 Mbp on chromosome 7, and Ghd8 at 4.3 Mbp on chromosome 8. A few loci are within 500 Kbp of genes/QTLs, being D88 at 5.4 Mbp on chromosome 3, GS5 at 3.4 Mbp on chromosome 5, and both qGL7.2 at 24.6–9 and DEP2 at 25.2 Mbp on chromosome 7. These known genes only account for a small proportion of the identified QTLs for grain mass in this study, indicating that there a large number of novel QTLs detected within this population for grain mass. A grain yield QTL detected by Liang et al. (2016a) at 22.6 Mbp on chromosome 6 co-localized with the QTL detected here in Madhupur 2014.

For traits straw biomass and harvest index, relatively few reports of QTL mapping, especially using GWAS are available, and few genes are known. None the less, the co-localization of a small number of the QTLs detected here with previous QTLs were found (**Supplementary Tables S1**, **S2**). One gene affecting harvest index, *APO1* identified by Terao et al. (2010), localizes with a weak QTL detected here only in AWD in Mn year 2.

While the flowering time was deliberately constrained due to the strategic development of the BAAP, there was still variation that allowed for successful GWA mapping. Since a number of the genes that regulate flowering time have been identified, this trait can be used to assess the effectiveness of the GWA mapping in this population. The GWA mapping of flowering time highlighted a large number of loci that control flowering time. Some of these loci were only detected in a single experiment, while a number were detected in more than one study and a single loci was detected in three of the four environments. As flowering time was measured on only a single replicate in each environment and there are only two experiments in which it was measured (Mymensingh year 1 and 2), it is not possible at this stage to state if any of the loci detected in the AWD experiments are specific to one treatment. Further investigation is needed to support this hypothesis. However, there is some indication that loci could be AWD specific [e.g., see comparison with Volante et al. (2017) study above at about 27 Mbp on chromosome 4 and an association around 2.5 Mbp on chromosome 11 which co-localized with *RCN*, a gene believed to be involved in flowering in rice (Nakagawa et al., 2002)]. The gene network that regulates flowering in rice has been studied in detail (Lee and An, 2015) with a number of the key genes cloned [e.g., Hd1 (Yano et al., 2000), Hd3 (Monna et al., 2002), and DTH8 (Wei et al., 2010)]. For more examples, see Lee and An (2015). In this study, eight genomic regions where significant SNPs (across both treatments and year) were detected have previously been identified as regions containing genes known to be involved in flowering time (**Table 6**). While a number of associations co-localize with known genes, a large number of associations do not, and further study of these is needed to ascertain their function. There are a number of reasons that might explain why known flowering time genes were not detected as flowering time QTLs in this population. Most important, the narrow flowering time window imposed on the population will mean that the different genotypes are likely to share similar alleles for the major flowering time genes in rice.

## Conclusion

In conclusion, the BAAP population is an excellent tool for the determination of yield traits, showing a good range of phenotypic variation and the detection of highly reproducible QTLs. It should prove valuable for other traits where a narrow window of flowering is important such as grain quality traits. There are several QTLs which should be targets for further study due to the detection of the loci in multiple experiments over years and sites.

## Author Contributions

AP and GN selected the cultivars for the population. GN, AP, AT, MS, and MI designed the field experiments. MS and MI supervised the field experiments. AT and AD developed the GWAS pipeline and conducted the GWA mapping. SF and EDPA processed and analyzed the genomic data and produced the SNP database. PR-a conducted the analysis of the flowering QTLs. MN extracted the rice DNA. KM provided support in the development of the SNP database, the selection of cultivars and managed the multiplication and distribution of the seed. AP, AT, and GN interpreted the data. AP and GN wrote the first draft of the manuscript. All authors reviewed the manuscript and approved the content of this manuscript.

## Conflict of Interest Statement

The authors declare that the research was conducted in the absence of any commercial or financial relationships that could be construed as a potential conflict of interest.

## Footnotes

1 http://www.bioinformatics.babraham.ac.uk/projects/trim_galore/

2 ftp://ftp.ensemblgenomes.org/pub/plants/release-21/fasta/oryza_sativa/dna/

3 http://broadinstitute.github.io/picard/

4 http://oryzasnp-atcg-irri-org.s3-website-ap-southeast-1.amazonaws.com/

5 http://snp-seek.irri.org/

6 https://doi.org/10.7910/DVN/AUMTIH

7 https://github.com/tony-travis/PIQUE

## Supplementary Material

FIGURE S1 Evanno plot of Delta-K from STRUCTURE analysis using 326 SNP markers on the 266 *aus* accessions of the BAAP.

FIGURE S2 fastSTRUCTURE analysis on the BAAP using 2,053,863 SNPs.

FIGURE S3 ADMIXTURE analysis of the BAAP using 2,053,863 SNPs.

FIGURE S4 Grain mass at Mymensingh in 2013 **(a)**, 2014 **(c)**, and Madhupur 2014 **(e)**. Correlation between AWD and CF at Mymensingh in 2013 **(b)**, 2014 **(d)**, and Madhupur 2014 **(f)**. Dashed lines in panels **(b,d,f)** are the regression lines for the traits. Solid line in panels **(b,d,f)** are the 1:1 lines. Points are the mean of three or four replicates.

FIGURE S5 Straw biomass at Mymensingh in 2013 **(a)**, 2014 **(c)**, and Madhupur 2014 **(e)**. Correlation between AWD and CF at Mymensingh in 2013 **(b)**, 2014 **(d)**, and Madhupur 2014 **(f)**. Dashed lines in panels **(b,d,f)** are the regression lines for the traits. Solid line in panels **(b,d,f)** are the 1:1 lines. Points are the mean of three or four replicates.

FIGURE S6 Harvest index at Mymensingh in 2013 **(a)**, 2014 **(c)**, and Madhupur 2014 **(e)**. Correlation between AWD and CF at Mymensingh in 2013 **(b)**, 2014 **(d)**, and Madhupur 2014 **(f)**. Dashed lines in panels **(b,d,f)** are the regression lines for the traits. Solid line in panels **(b,d,f)** are the 1:1 lines. Points are the mean of three or four replicates.

FIGURE S7 Flowering time index at Mymensingh in 2013 **(a)**, and 2014 **(c)**. Correlation between AWD and CF at Mymensingh in 2013 **(b)**, and 2014 **(d)**. Dashed lines in panels **(b,d)** are the regression lines for the traits. Solid line in panels **(b,d)** are the 1:1 lines. Correlation analysis across the different sites for flowering time across both year under the same treatment [AWD **(e)** and CF **(f)**]. Correlation analysis performed on 191 accessions that were represented (*n* ≥ 3) at all field sites under both treatments, the solid line in these graphs is the regression line. All correlation are significant (*P* < 0.001). Points are the mean of three or four replicates.

FIGURE S8 Correlation analysis across the different sites for grain mass. Correlation analysis performed on 191 accessions that were represented (*n* ≥ 3) at all field sites under both treatments. All correlation are significant (*P* < 0.001). Points are the mean of three or four replicates.

FIGURE S9 Correlation analysis across the different sites for straw biomass. Correlation analysis performed on 191 accessions that were represented (*n* ≥ 3) at all field sites under both treatments. All correlation are significant (*P* < 0.001). Points are the mean of three or four replicates.

FIGURE S10 Correlation analysis across the different sites for harvest index. Correlation analysis performed on 191 accessions that were represented (*n* ≥ 3) at all field sites under both treatments. All correlation are significant (*P* < 0.001). Points are the mean of three or four replicates.

FIGURE S11 Manhattan plots from GWAS straw biomass (for details, see **Figure 6**).

FIGURE S12 Manhattan plots from GWAS harvest index (for details, see **Figure 6**).

TABLE S1 Notable association detected for Straw Biomass.

TABLE S2 Notable association detected for Harvest Index.

FILE S1 The BAAP accession details, sequence metadata, subgroup attribution, and phenotype data.

FILE S2 SNPs associated with grain yield including chromosome graphs.

FILE S3 SNPs associated with straw biomass including chromosome graphs.

FILE S4 SNPs associated with harvest index including chromosome graphs.

FILE S5 SNPs associated with flowering time including chromosome graphs.
