# Supplementary material for: Genome Wide Association Mapping of Grain and Straw Biomass Traits in the Rice Bengal and Assam Aus Panel (BAAP) Grown Under Alternate Wetting and Drying and Permanently Flooded Irrigation
Source: Front Plant Sci. 2018 Sep 3;9:1223. doi: 10.3389/fpls.2018.01223 (PMC6129953; doi:10.3389/fpls.2018.01223)
Supplement: FIGURE S1 — Evanno plot of Delta-K from STRUCTURE analysis using 326 SNP markers on the 266 aus accessions of the BAAP. [file Presentation_1.pptx]

## Slide 1
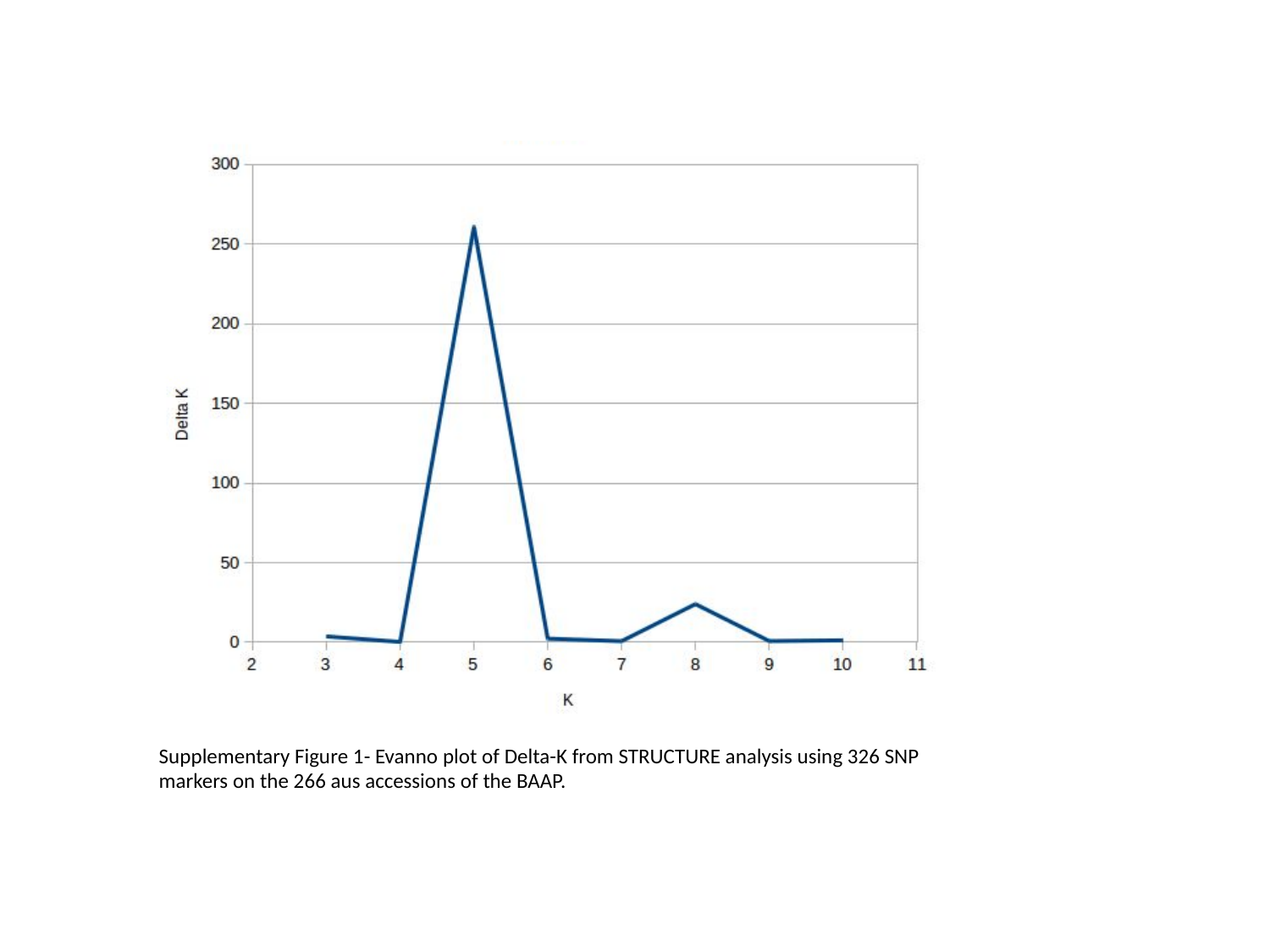

Supplementary Figure 1- Evanno plot of Delta-K from STRUCTURE analysis using 326 SNP markers on the 266 aus accessions of the BAAP.

## Slide 2
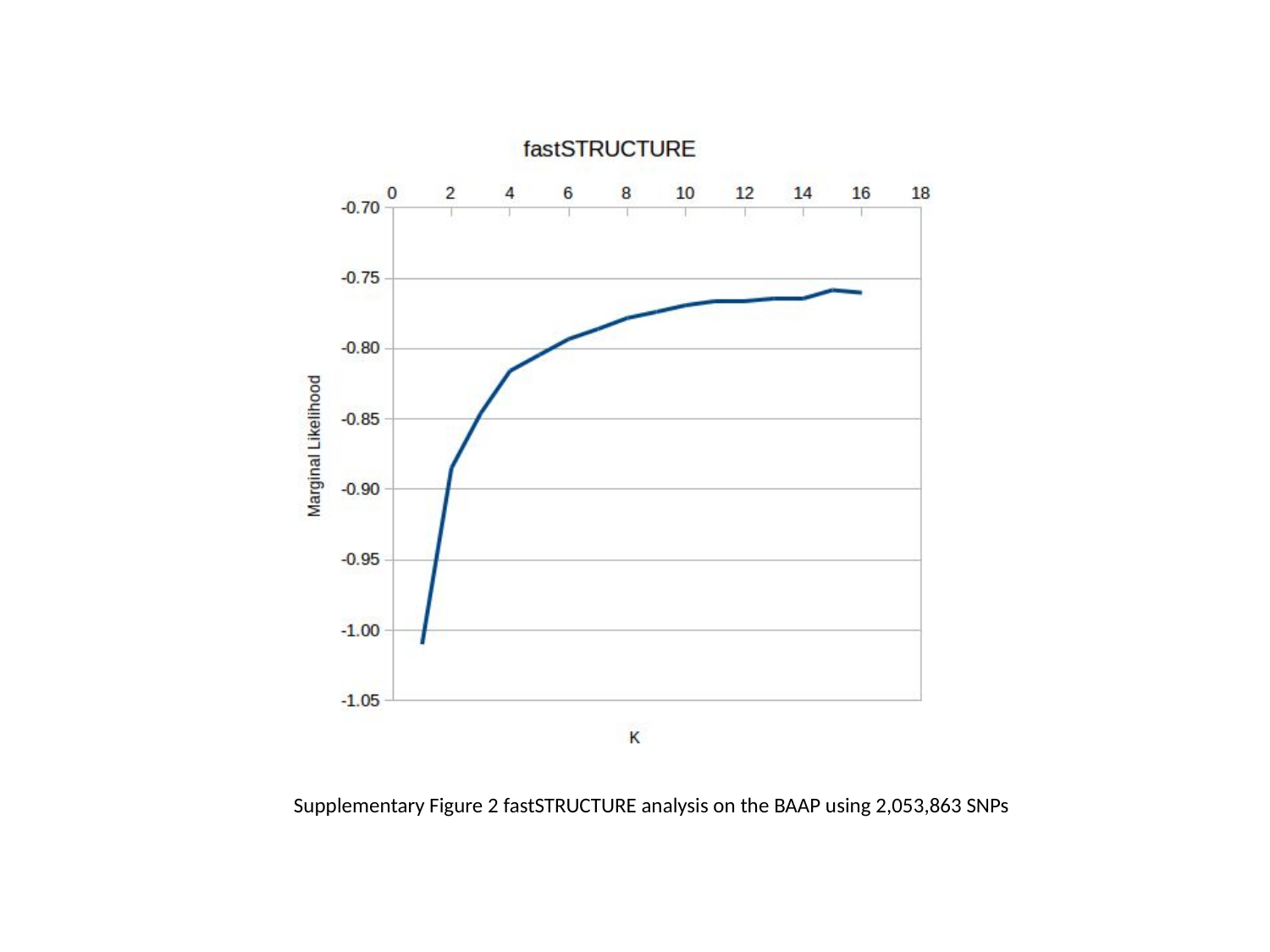

Supplementary Figure 2 fastSTRUCTURE analysis on the BAAP using 2,053,863 SNPs

## Slide 3
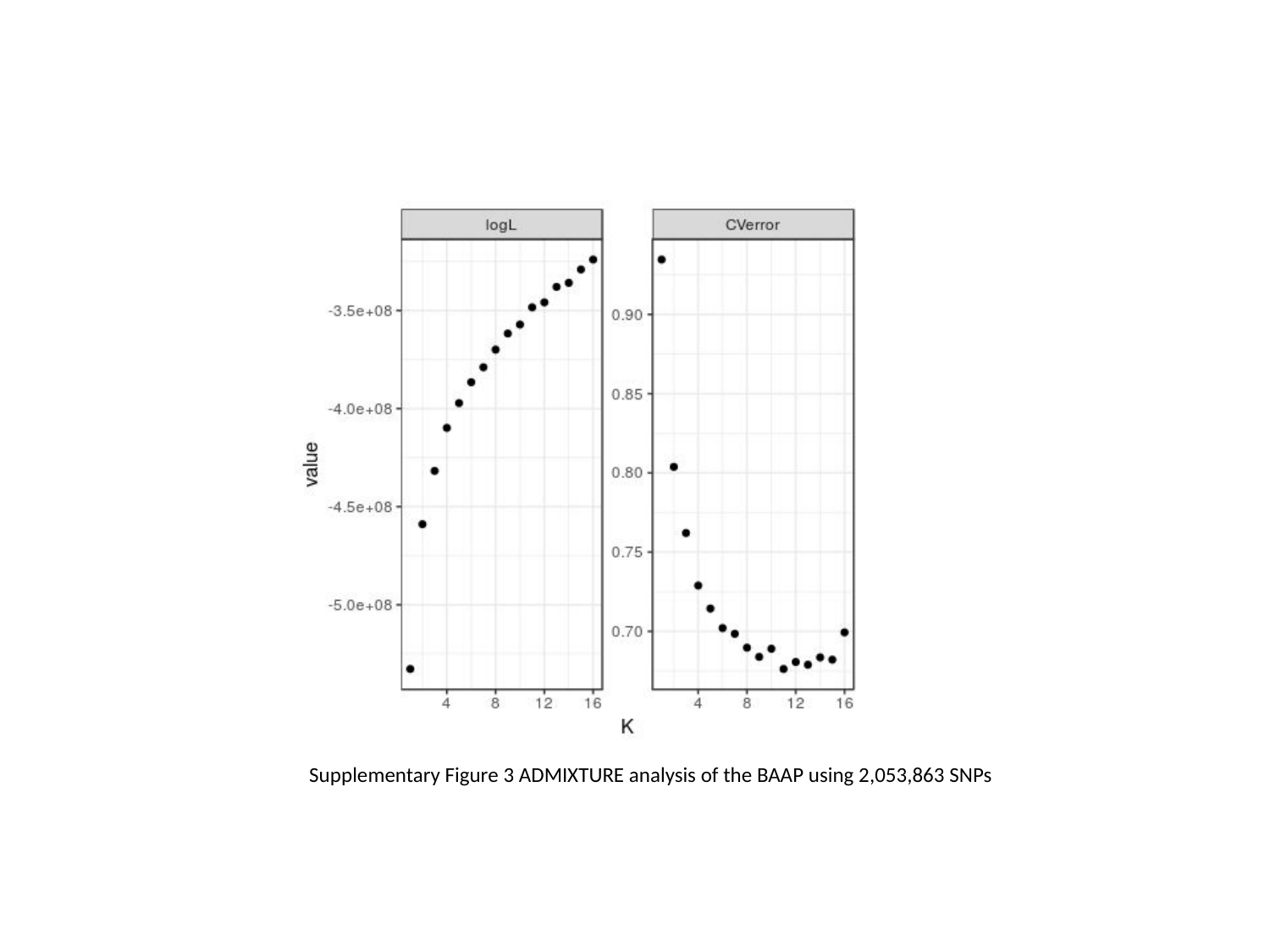

Supplementary Figure 3 ADMIXTURE analysis of the BAAP using 2,053,863 SNPs

## Slide 4
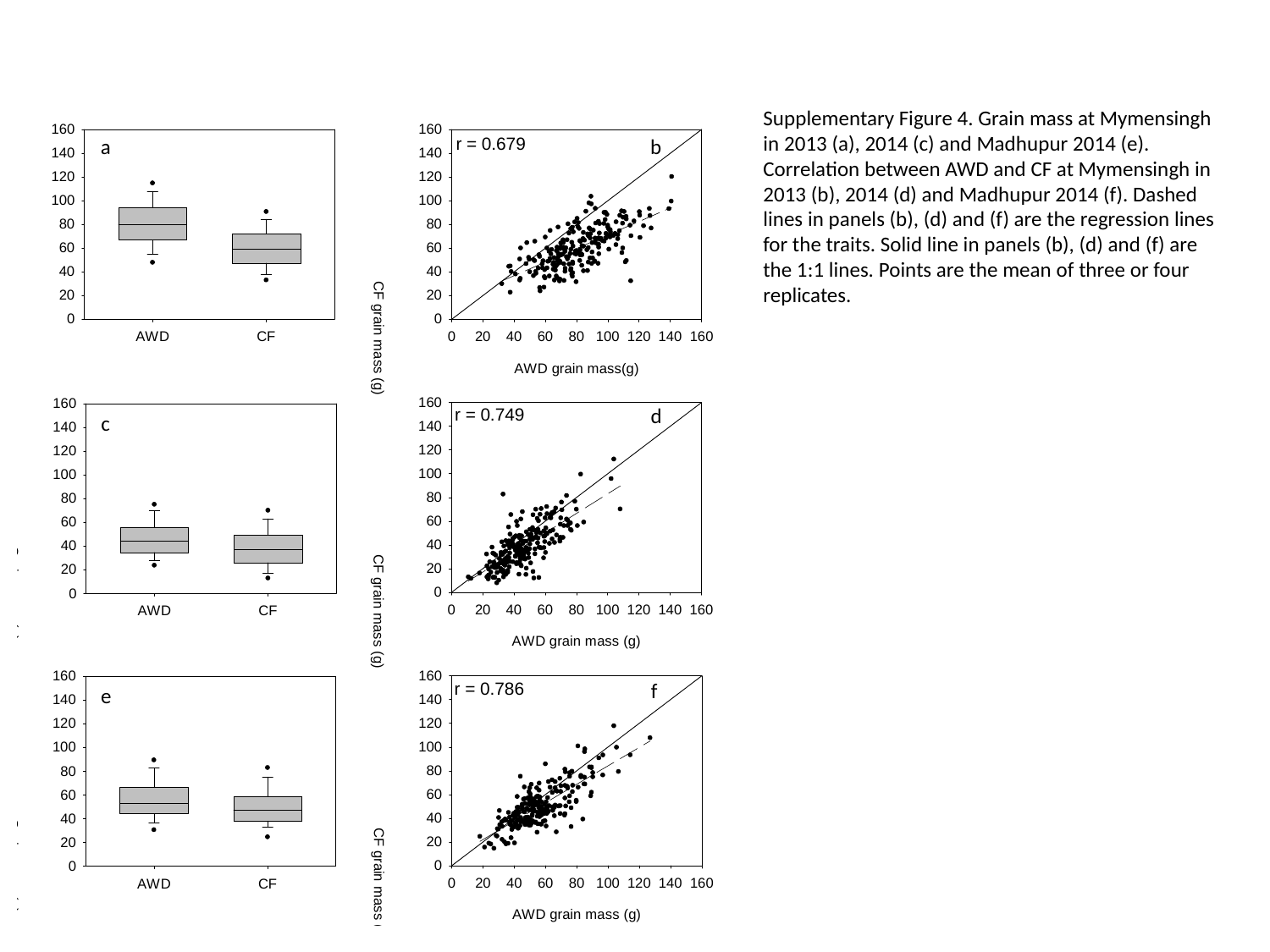

Supplementary Figure 4. Grain mass at Mymensingh in 2013 (a), 2014 (c) and Madhupur 2014 (e). Correlation between AWD and CF at Mymensingh in 2013 (b), 2014 (d) and Madhupur 2014 (f). Dashed lines in panels (b), (d) and (f) are the regression lines for the traits. Solid line in panels (b), (d) and (f) are the 1:1 lines. Points are the mean of three or four replicates.
b
a
d
c
f
e

## Slide 5
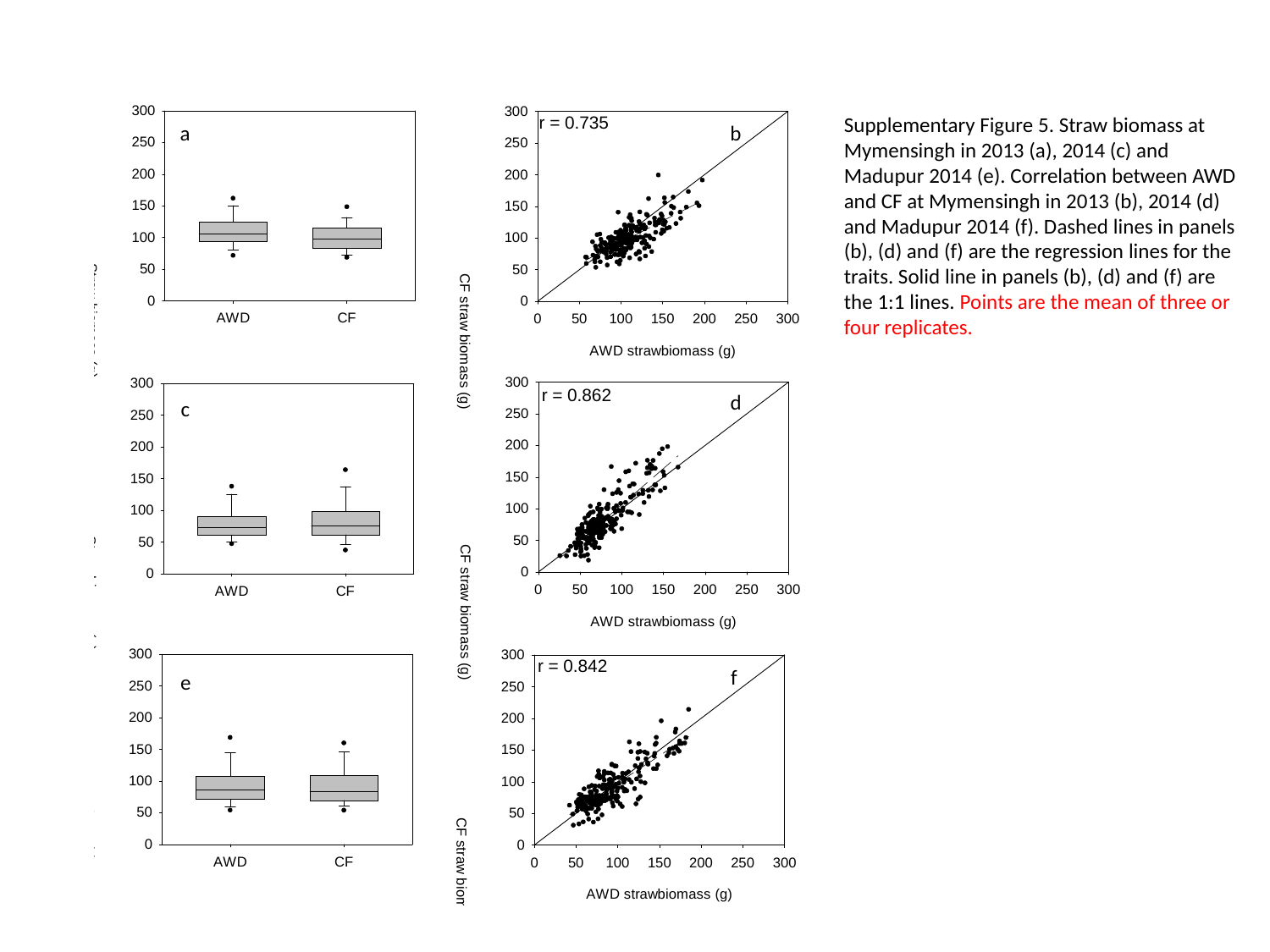

Supplementary Figure 5. Straw biomass at Mymensingh in 2013 (a), 2014 (c) and Madupur 2014 (e). Correlation between AWD and CF at Mymensingh in 2013 (b), 2014 (d) and Madupur 2014 (f). Dashed lines in panels (b), (d) and (f) are the regression lines for the traits. Solid line in panels (b), (d) and (f) are the 1:1 lines. Points are the mean of three or four replicates.
b
a
d
c
f
e

## Slide 6
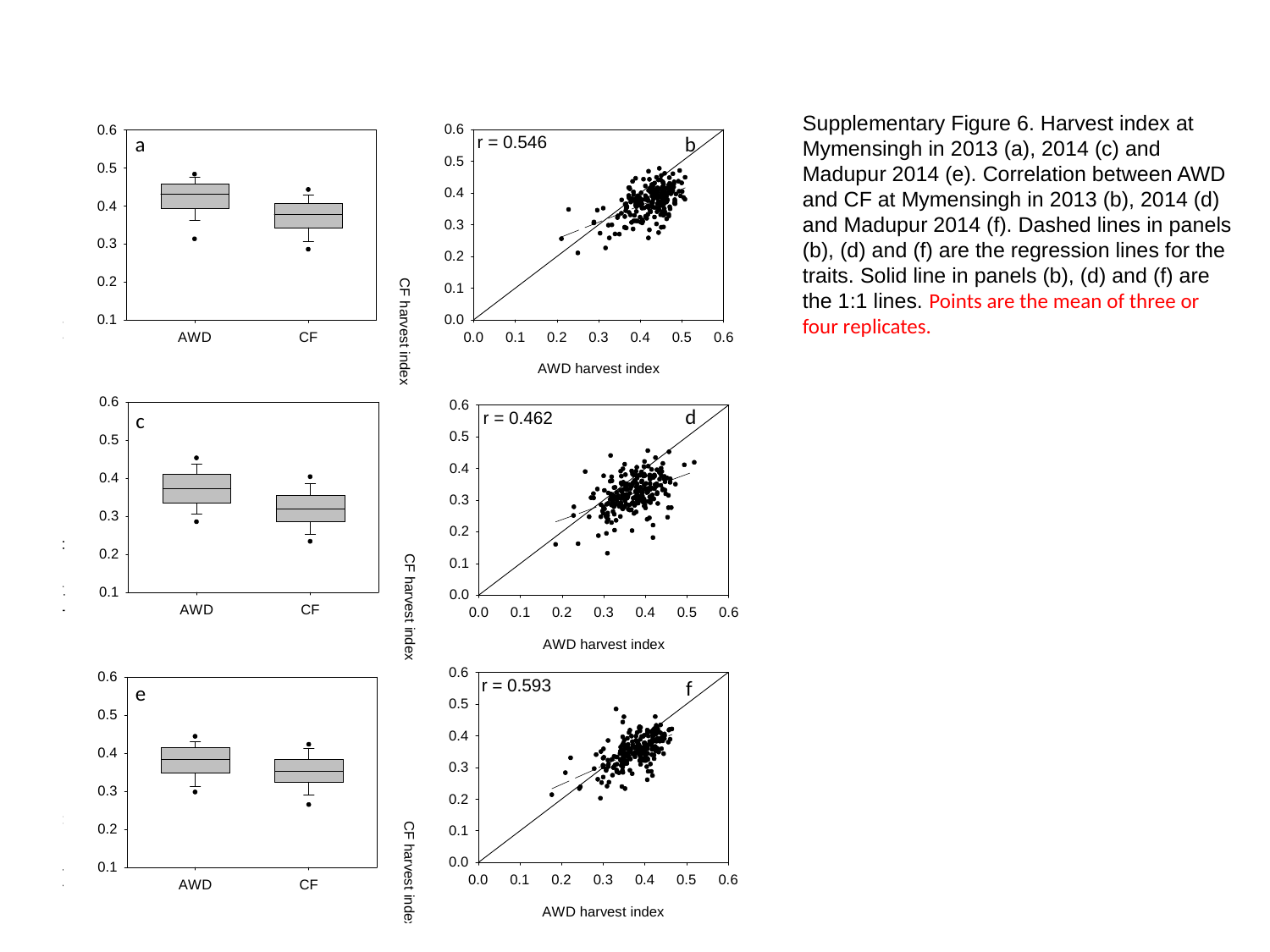

Supplementary Figure 6. Harvest index at Mymensingh in 2013 (a), 2014 (c) and Madupur 2014 (e). Correlation between AWD and CF at Mymensingh in 2013 (b), 2014 (d) and Madupur 2014 (f). Dashed lines in panels (b), (d) and (f) are the regression lines for the traits. Solid line in panels (b), (d) and (f) are the 1:1 lines. Points are the mean of three or four replicates.
b
a
d
c
f
e

## Slide 7
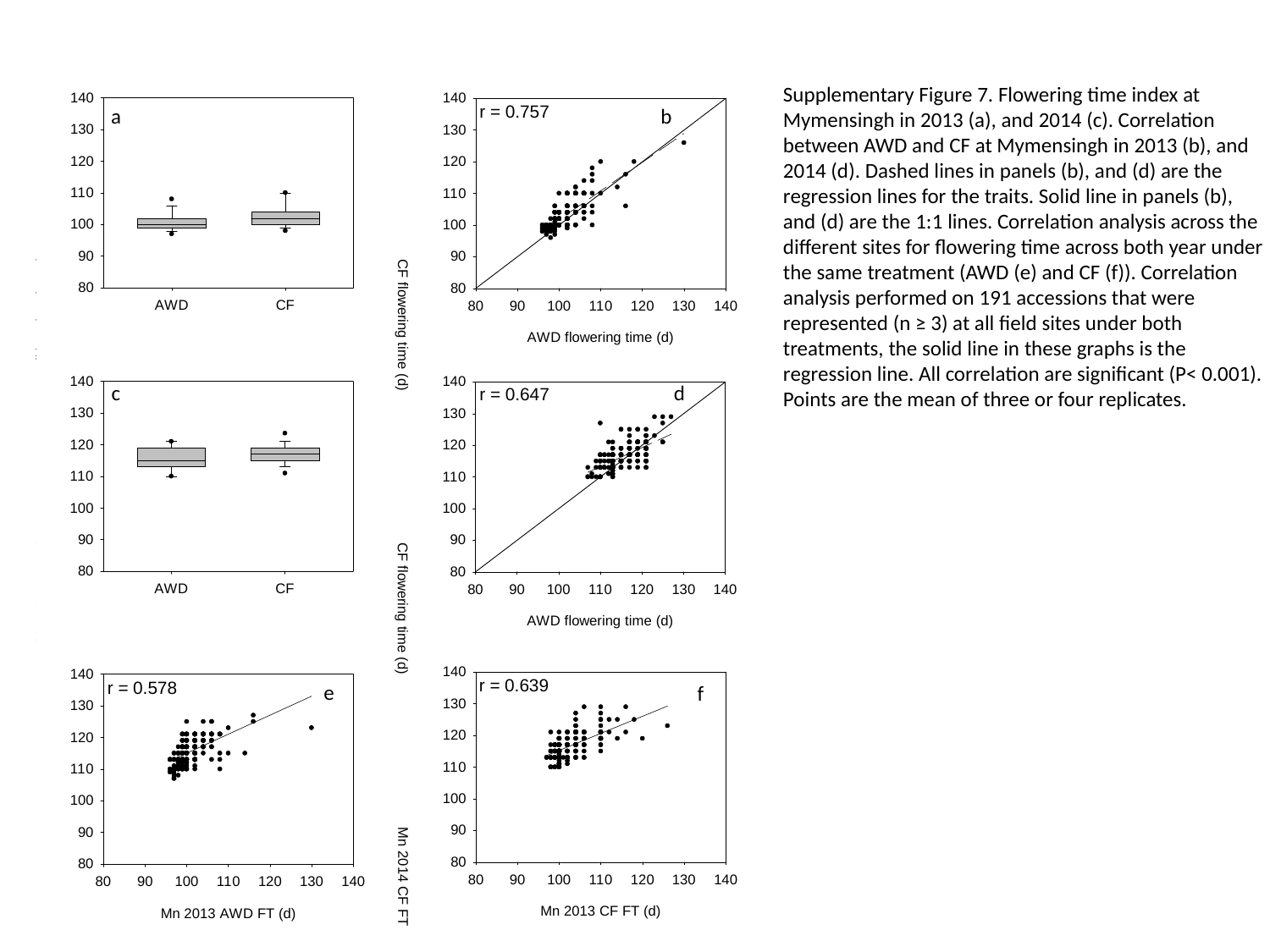

Supplementary Figure 7. Flowering time index at Mymensingh in 2013 (a), and 2014 (c). Correlation between AWD and CF at Mymensingh in 2013 (b), and 2014 (d). Dashed lines in panels (b), and (d) are the regression lines for the traits. Solid line in panels (b), and (d) are the 1:1 lines. Correlation analysis across the different sites for flowering time across both year under the same treatment (AWD (e) and CF (f)). Correlation analysis performed on 191 accessions that were represented (n ≥ 3) at all field sites under both treatments, the solid line in these graphs is the regression line. All correlation are significant (P< 0.001). Points are the mean of three or four replicates.
b
a
d
c
e
f

## Slide 8
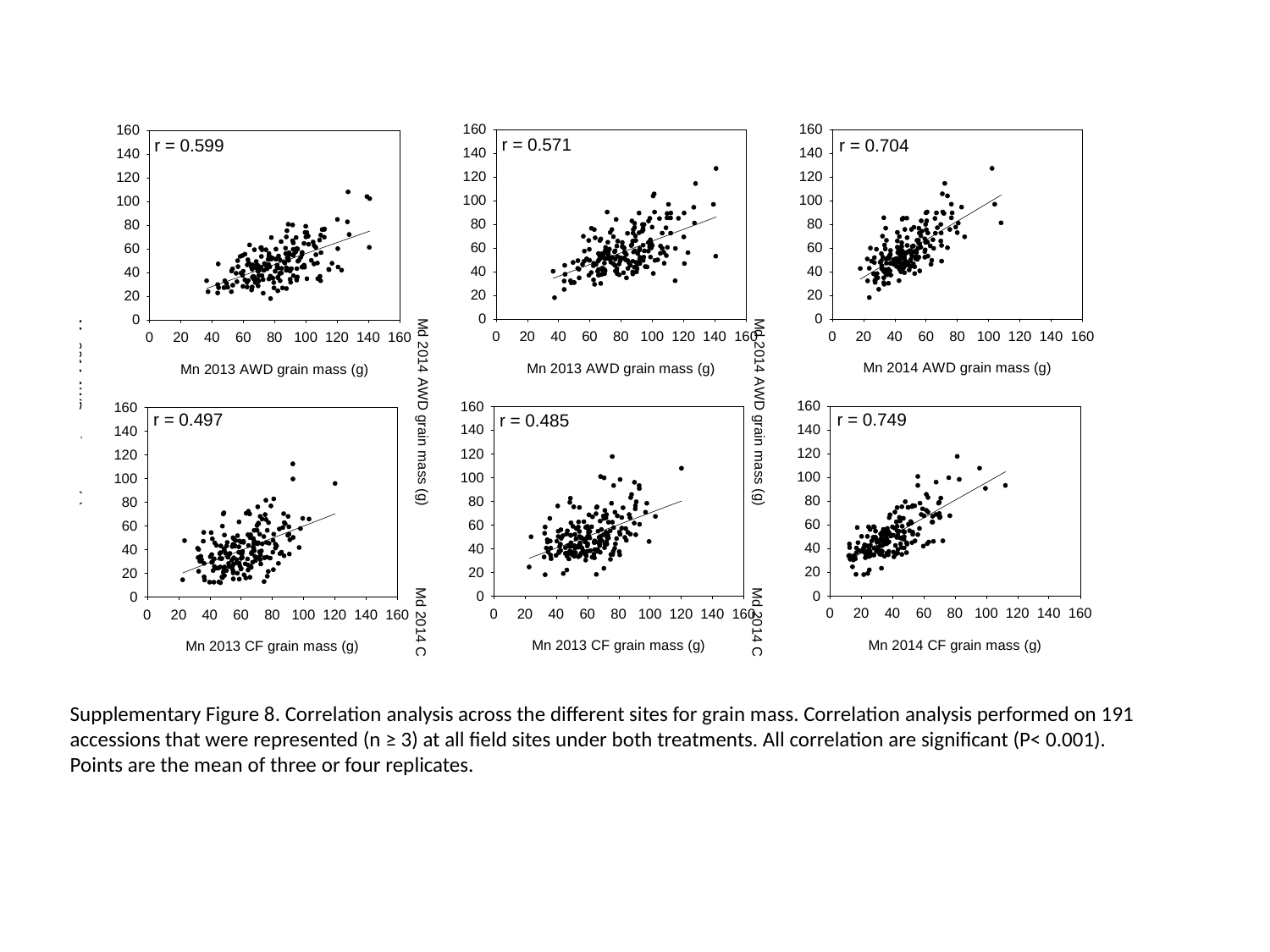

Supplementary Figure 8. Correlation analysis across the different sites for grain mass. Correlation analysis performed on 191 accessions that were represented (n ≥ 3) at all field sites under both treatments. All correlation are significant (P< 0.001). Points are the mean of three or four replicates.

## Slide 9
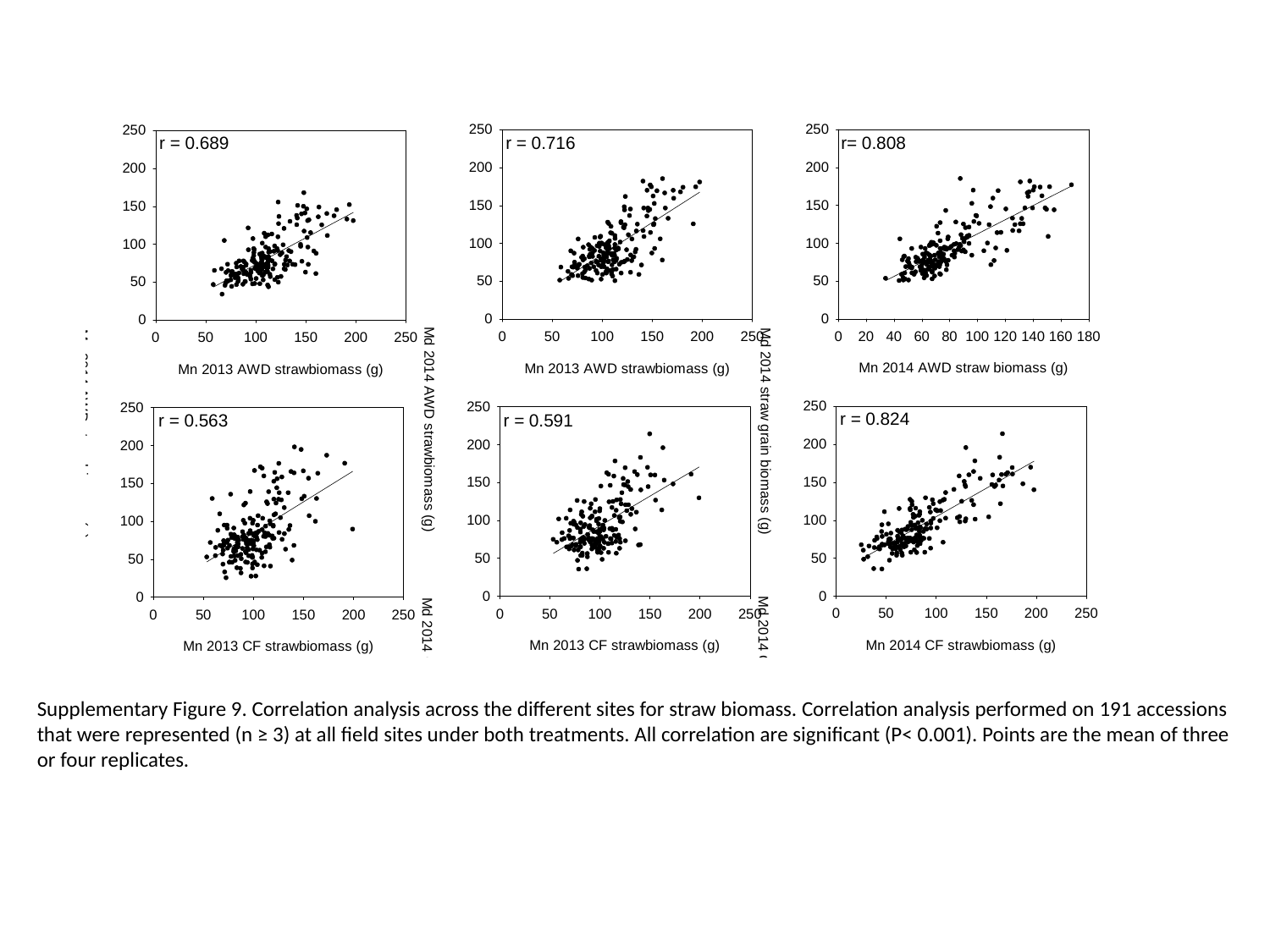

Supplementary Figure 9. Correlation analysis across the different sites for straw biomass. Correlation analysis performed on 191 accessions that were represented (n ≥ 3) at all field sites under both treatments. All correlation are significant (P< 0.001). Points are the mean of three or four replicates.

## Slide 10
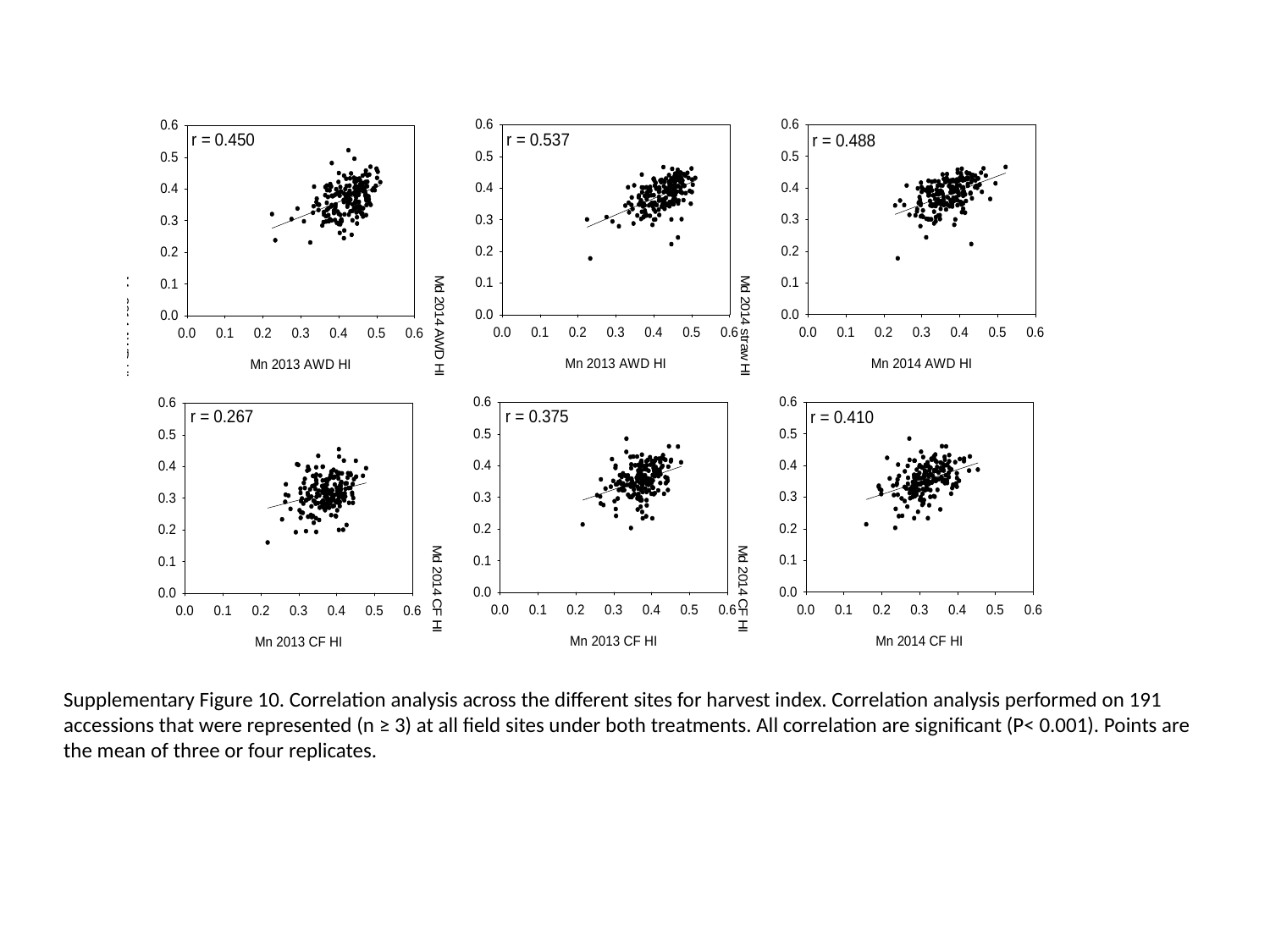

Supplementary Figure 10. Correlation analysis across the different sites for harvest index. Correlation analysis performed on 191 accessions that were represented (n ≥ 3) at all field sites under both treatments. All correlation are significant (P< 0.001). Points are the mean of three or four replicates.

## Slide 11
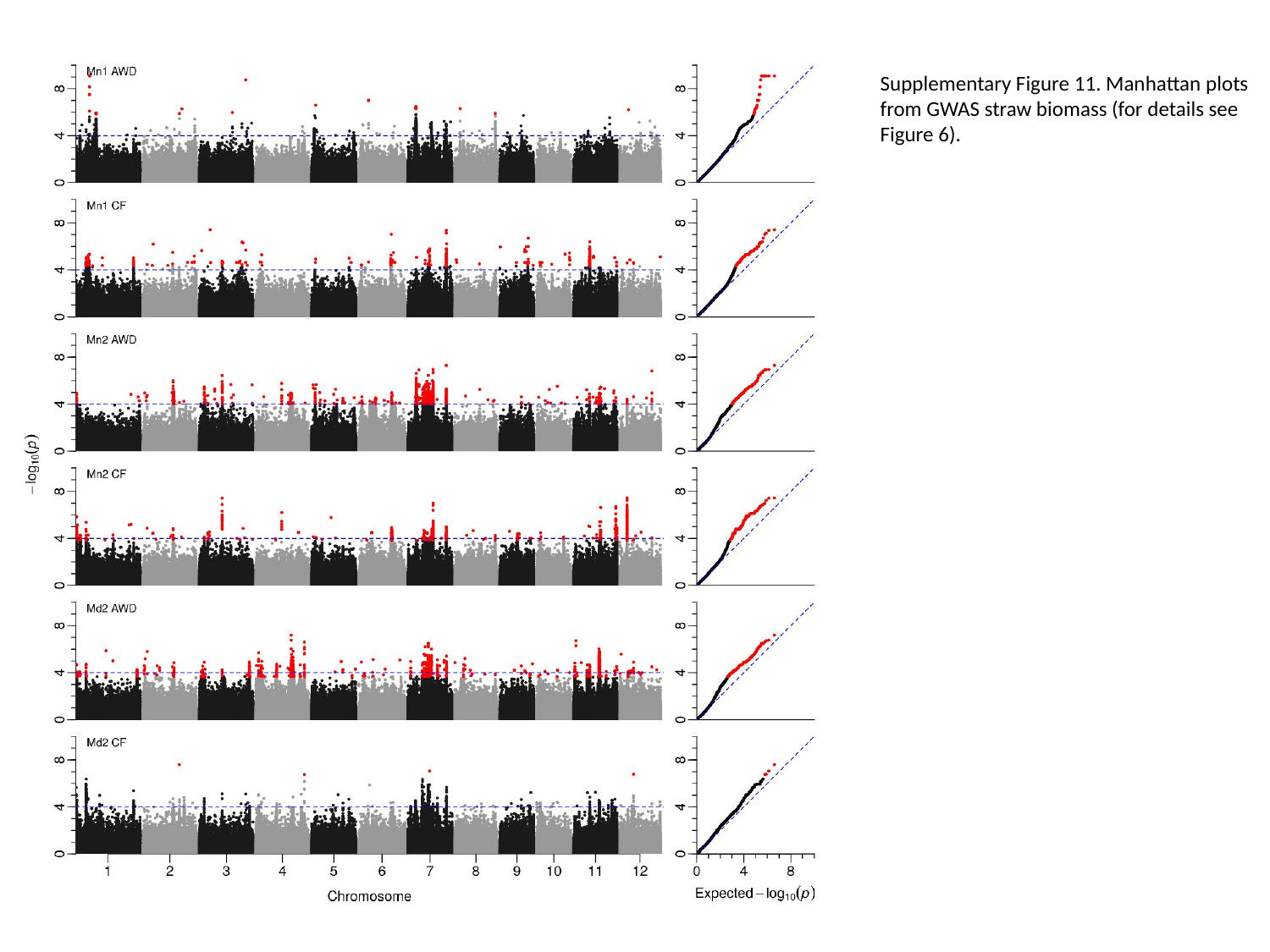

Supplementary Figure 11. Manhattan plots from GWAS straw biomass (for details see Figure 6).

## Slide 12
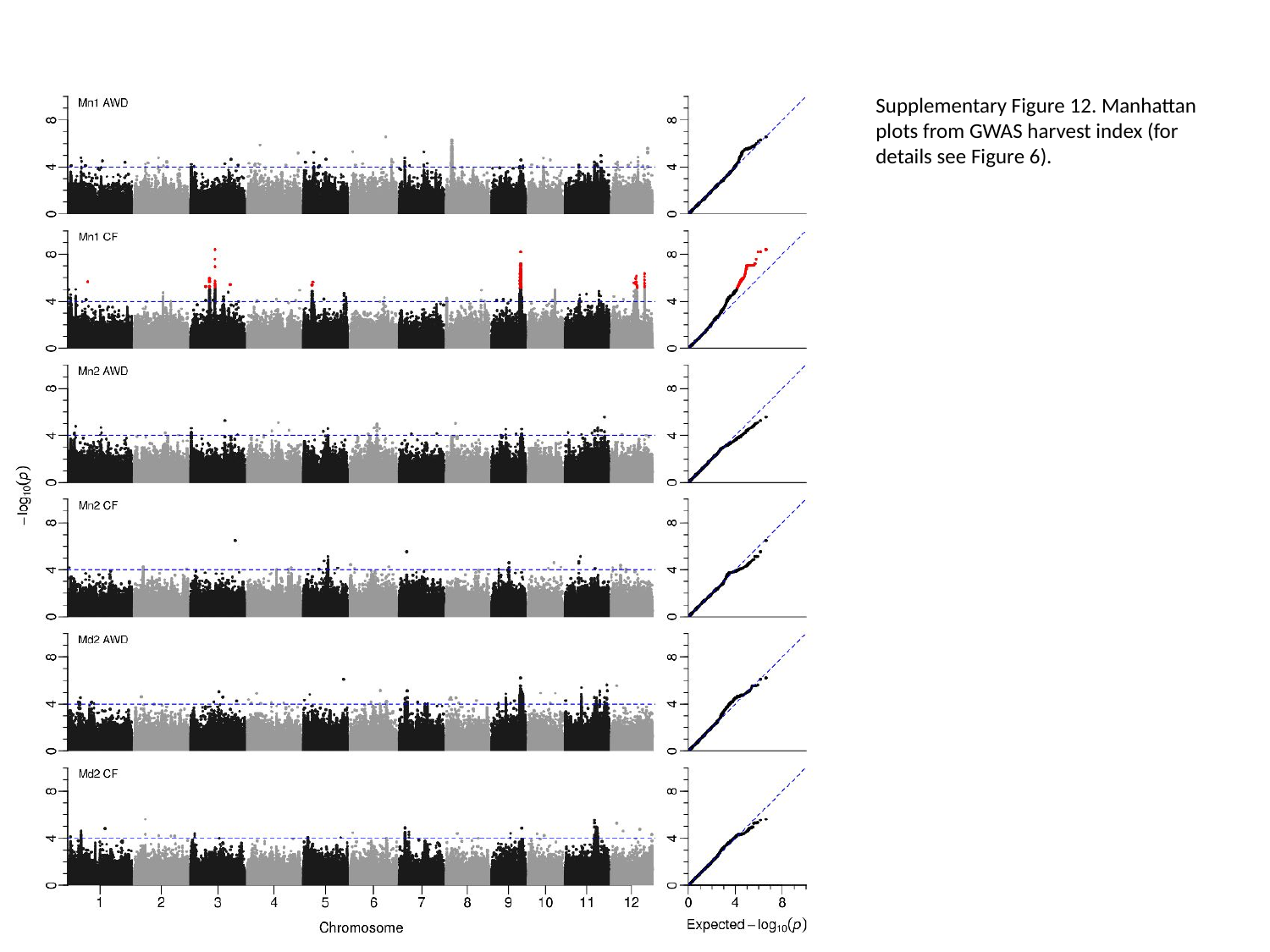

Supplementary Figure 12. Manhattan plots from GWAS harvest index (for details see Figure 6).
