# Supplementary material for: Genome Wide Association Mapping of Grain and Straw Biomass Traits in the Rice Bengal and Assam Aus Panel (BAAP) Grown Under Alternate Wetting and Drying and Permanently Flooded Irrigation
Source: Front Plant Sci. 2018 Sep 3;9:1223. doi: 10.3389/fpls.2018.01223 (PMC6129953; doi:10.3389/fpls.2018.01223)
Supplement: TABLE S1 — Notable association detected for Straw Biomass. [file Table_1.DOCX]

Supplementary table 1. Notable association detected for Biomass

| Chromo-  some | Position (Mbp) | Number of significant SNPs detected | | | | | | Previously detected  QTL |
| --- | --- | --- | --- | --- | --- | --- | --- | --- |
|  |  | Mn Year 1 | | Mn Year 2 | | Md Year 2 | |  |
|  |  | AWD | CF | AWD | CF | AWD | CF |  |
| 1 | 0.01-0.72 |  |  | 26 | 92 | 17 | 28 | a |
| 1 | 6.65-7.02 | 2 | 78 |  | 3 | 3 | 82 |  |
| 1 | 13.51-13.57 | 85 |  |  |  |  |  |  |
| 1 | 38.50-38.69 |  | 33 |  |  |  | 21 | b,c |
| 1 | 41.12-41.76 |  |  | 1 |  | 9 |  |  |
| 2 | 19.39-20.73 |  | 6 | 121 | 8 | 7 | 4 |  |
| 2 | 24.22-24.49 | 1 | 3 | 1 |  |  | 3 |  |
| 2 | 34.52-34.74 | 3 | 1 |  |  |  |  |  |
| 3 | 1.22-1.23 |  |  |  |  | 6 |  |  |
| 3 | 2.37-3.47 |  |  | 13 | 3 | 6 | 6 |  |
| 3 | 4.02-4.04 |  |  | 3 |  | 1 |  |  |
| 3 | 5.87-5.90 |  | 1 | 2 | 5 |  |  |  |
| 3 | 15.39-15.48 |  |  |  | 32 | 4 | 2 |  |
| 3 | 28.62 | 2 | 4 |  |  |  |  |  |
| 3 | 33.61-33.80 |  |  |  |  | 38 | 2 |  |
| 4 | 1.78-1.95 |  |  |  |  | 19 | 5 |  |
| 4 | 3.59-4.00 |  | 3 |  |  | 7 | 2 |  |
| 4 | 13.56-13.96 |  |  |  |  | 11 | 4 |  |
| 4 | 17.12-17.23 |  |  | 6 | 5 |  |  | c |
| 4 | 23.06-23.08 |  |  | 45 |  |  |  | c |
| 4 | 23.59-25.45 |  |  | 6 |  | 1076 | 1 |  |
| 4 | 32.52-32.95 | 1 |  | 1 |  | 11 | 7 |  |
| 5 | 2.45-2.46 |  |  | 17 |  |  |  |  |
| 5 | 21.11-21.11 |  |  | 10 |  | 2 | 1 |  |
| 5 | 24.97-25.16 |  | 3 |  |  |  | 1 | b |
| 6 | 1.82-1.85 |  | 12 |  |  | 1 |  |  |
| 6 | 2.12-2.13 |  |  | 18 |  |  |  |  |
| 6 | 11.07-11.40 |  |  | 13 |  |  |  |  |
| 6 | 21.84-22.68 |  | 1 | 21 | 111 |  |  |  |
| 7 | 1.82-1.83 |  |  |  |  | 7 | 1 | b |
| 7 | 4.85-5.68 | 438 | 1 | 561 |  |  | 15 | b |
| 7 | 7.31-7.33 |  |  | 8 |  |  |  | b |
| 7 | 9.35-10.35 |  | 1 | 9 | 1 | 5 | 37 |  |
| 7 | 10.56-14.12 |  | 14 | 204 | 22 | 103 | 50 |  |
| 7 | 14.49-15.19 | 1 | 16 | 17 | 2 | 13 | 12 |  |
| 7 | 15.77-16.93 | 24 |  | 252 | 269 | 24 | 2 |  |
| 7 | 19.64-19.78 |  |  | 1 |  | 39 | 15 |  |
| 7 | 24.04-24.39 | 3 | 1 |  |  | 3 |  |  |
| 7 | 25.54-25.84 | 44 | 261 | 231 | 47 | 27 | 85 |  |
| 8 | 5.98-6.02 |  |  |  |  | 7 |  |  |
| 8 | 6.75 |  |  |  |  | 4 | 2 |  |
| 8 | 25.77-25.83 |  |  | 1 |  |  | 3 |  |
| 8 | 27.11-27.27 | 71 |  |  | 2 |  |  |  |
| 9 | 11.84-11.94 |  |  |  | 9 |  |  |  |
| 9 | 14.42-14.62 | 1 |  | 4 |  | 2 | 3 |  |
| 10 | 22.06-22.22 |  | 5 |  |  | 1 |  |  |
| 11 | 0.73-0.78 |  |  |  |  | 10 |  |  |
| 11 | 10.37-10.72 |  | 605 | 5 | 1 | 5 | 5 |  |
| 11 | 16.83-17.47 |  | 1 | 277 | 3 | 637 | 16 |  |
| 11 | 17.63-18.26 |  | 5 | 6 | 13 |  |  |  |
| 11 | 24.12-24.27 | 7 |  |  |  |  |  |  |
| 11 | 25.55-25.58 |  |  |  |  |  | 10 |  |
| 11 | 27.69-27.70 |  |  |  | 1 | 3 | 1 |  |
| 11 | 28.12-28.14 |  |  | 3 | 27 |  |  |  |
| 11 | 28.66-28.69 |  | 2 |  |  | 1 |  |  |
| 11 | 28.94-29.00 |  |  |  |  | 6 | 1 |  |
| 12 | 4.79-5.13 |  | 1 | 5 | 1527 |  |  |  |
| 12 | 9.35 |  | 5 | 1 |  | 4 | 11 |  |
| 12 | 21.61 |  |  | 13 | 4 | 4 | 3 |  |

a = Bhattarai and Subudhi 2018, b = ,Suji et al., 2012, c = Liu et al., 2006.
