# Supplementary material for: Genome Wide Association Mapping of Grain and Straw Biomass Traits in the Rice Bengal and Assam Aus Panel (BAAP) Grown Under Alternate Wetting and Drying and Permanently Flooded Irrigation
Source: Front Plant Sci. 2018 Sep 3;9:1223. doi: 10.3389/fpls.2018.01223 (PMC6129953; doi:10.3389/fpls.2018.01223)
Supplement: TABLE S2 — Notable association detected for Harvest Index. [file Table_2.DOCX]

Supplementary table 2. Notable association detected for Harvest Index

| Chromo-  some | Position (Mbp) | Number of significant SNPs detected | | | | | | Previously detected? | | |
| --- | --- | --- | --- | --- | --- | --- | --- | --- | --- | --- |
|  |  | Mn Year 1 | | Mn Year 2 | | Md Year 2 | | GWAS | QTL | Gene |
|  |  | AWD | CF | AWD | CF | AWD | CF |  |  |  |
| 1 | 2.17-2.24 |  | 3 |  |  |  | 1 |  |  |  |
| 1 | 4.64-4.65 |  |  | 9 |  |  |  |  |  |  |
| 1 | 5.49-5.55 |  | 8 | 1 |  |  |  |  |  |  |
| 1 | 9.11-9.21 | 1 |  |  |  |  | 42 |  |  |  |
| 2 | 5.76 |  |  |  | 5 |  |  |  |  |  |
| 2 | 7.23 | 1 |  |  |  |  | 2 | a |  |  |
| 2 | 19.17-19.20 |  | 62 |  |  |  |  |  |  |  |
| 3 | 0.48-0.49 | 1 |  | 6 |  |  |  |  |  |  |
| 3 | 12.62-12.82 |  | 20 |  |  |  |  |  | b |  |
| 3 | 15.95-16.32 |  | 239 |  |  |  |  |  |  |  |
| 5 | 5.99-6.22 |  | 7 |  |  |  |  |  |  |  |
| 5 | 16.33-16.86 |  |  | 3 | 73 |  |  | a |  |  |
| 6 | 17.66-17.78 |  |  | 5 |  |  |  |  |  |  |
| 6 | 24.35-24.40 |  |  |  | 1 | 2 |  |  |  |  |
| 6 | 27.52-27.71 | 6 |  |  |  |  |  |  |  | APO1^c^ |
| 7 | 3.83-3.86 |  |  |  |  | 13 | 58 |  |  |  |
| 8 | 0.18-0.19 |  | 6 |  |  |  |  |  |  |  |
| 8 | 3.44-3.77 | 132 |  |  |  | 1 |  |  |  |  |
| 9 | 9.61-9.67 |  |  | 2 |  | 157 |  |  |  |  |
| 9 | 19.25-19.85 | 1 | 175 |  |  | 193 |  |  |  |  |
| 9 | 20.22-20.32 |  | 2 | 3 |  | 5 | 1 |  |  |  |
| 9 | 21.17-21.31 | 1 |  |  |  | 173 |  |  |  |  |
| 10 | 17.96-18.19 |  | 122 |  |  | 1 |  |  |  |  |
| 11 | 8.94 | 2 |  |  | 2 |  |  |  |  |  |
| 11 | 10.67-10.85 |  |  | 1 |  | 29 |  |  |  |  |
| 11 | 19.37-19.76 | 4 |  | 1 |  | 1 | 8 |  |  |  |
| 11 | 20.64-20.90 | 3 |  |  |  | 7 | 95 |  |  |  |
| 11 | 21.28-21.81 | 1 |  | 5 |  |  | 17 |  |  |  |
| 11 | 22.51-22.54 |  | 8 |  |  |  |  |  |  |  |
| 11 | 23.00-23.02 |  |  |  |  | 7 |  |  |  |  |
| 11 | 26.21-26.26 |  |  | 2 |  | 3 |  |  |  |  |
| 11 | 27.82-27.85 |  |  |  |  | 7 |  |  |  |  |
| 12 | 16.40-16.71 |  | 15 |  |  |  |  |  |  |  |
| 12 | 16.99-17.50 |  | 14 |  |  |  |  |  |  |  |
| 12 | 22.24-22.35 |  | 77 |  |  |  |  |  |  |  |

a = Guo et al., 2018, b = Hittalmani 2003 c = Terao et al., 2010
